# The Self-Organization of a Spoken Word

**DOI:** 10.3389/fpsyg.2012.00209

**Published:** 2012-07-06

**Authors:** John G. Holden, Srinivasan Rajaraman

**Affiliations:** ^1^Department of Psychology, CAP Center for Cognition, Action, and Perception, University of CincinnatiCincinnati, OH, USA

**Keywords:** response time distributions, inverse power law, self-organization, speeded word naming, interaction dominant dynamics, coordinative synergy, 1/f noise

## Abstract

Pronunciation time probability density and hazard functions from large speeded word naming data sets were assessed for empirical patterns consistent with multiplicative and reciprocal feedback dynamics – interaction dominant dynamics. Lognormal and inverse power law distributions are associated with multiplicative and interdependent dynamics in many natural systems. Mixtures of lognormal and inverse power law distributions offered better descriptions of the participant’s distributions than the ex-Gaussian or ex-Wald – alternatives corresponding to additive, superposed, component processes. The evidence for interaction dominant dynamics suggests fundamental links between the observed coordinative synergies that support speech production and the shapes of pronunciation time distributions.

Reading scientists concern themselves with the cognitive activity governing the translation of printed words into meaningful language. Speech production scientists seek a coherent description of the processes that guide production in speaking aloud. Traditionally, both these dimensions of reading aloud are studied as separate component process of cognition. Nevertheless, both aspects of performance are in play as an individual pronounces even a single visually presented word in a speeded naming experiment.

Speaking aloud, whether reading or in conversation, is a fundamentally dynamic, time varying activity. The simplest of utterances require speakers to adaptively coordinate an untidy throng of anatomical and physiological variables. Self-organizing coordinative synergies tame the inherent complexity of speaking, however (Turvey, 2007). This article describes how pronunciation times derived from a speeded naming task map into the coordinative speech activity along empirical and theoretical dimensions.

Historically, cognitive scientists used ideal probability distributions as models to explore cognitive performance. The most widely invoked working hypothesis was that a model’s parameters may selectively refer to the activity of specific cognitive processes (e.g., Townsend and Ashby, 1983; Luce, 1986). The term *component dominant dynamics* refers to this long-standing hypothesis in cognitive science: Behavioral measurements reflect the activity of isolable cognitive components, themselves, their time-course, and their functional details (e.g., Sternberg, 1969; Simon, 1973). A corollary assumption is that computational or symbolic cognitive processing operations occupy the bulk of the elapsed time between signal and response.

This article explores an alternate tack: The parameters of an ideal “cocktail” mixture distribution are hypothesized to collectively reflect the relative stability and flexibility of coordinative structures – the dynamics of the self-organized synergies that support reading and speaking aloud in speeded naming performance. The term interaction dominant dynamics codifies this alternative hypothesis: Behavioral measurements reflect emergent, irreducible, coordination and coupling among the processes that support the act of word naming.

Large samples of pronunciation times, obtained from many different individuals, are used to contrast the assumptions of the component dominant and interaction dominant accounts of the empirical patterns. As we describe next, speech production was shown, via independent sources of evidence, to be governed by self-organizing coordinative synergies. Coordinative synergies rely on interaction dominant dynamics. Since word naming entails a speech act, it offers a unique opportunity to directly asses how that coordinative activity is reflected in the shapes of pronunciation time distributions.

## Coordinative Synergies Behind the Spoken Word

Pronouncing-aloud an individual word may seem effortless and prosaic. It is well-established, however, that successfully uttering even a single phoneme requires a speaker to produce a complicated spatiotemporal arrangement among at least 70 muscles (Kelso et al., 1984, 1986; Turvey, 1990, 2007). Human speech presents an example of Bernstein’s (1967/1996) “degrees of freedom” problem; any theory of speech control that posits direct command of the relevant individual processes and variables is quickly overwhelmed and rendered implausible by the sheer number of processes that must be controlled. Coordinative synergies resolve the paradox. Synergies are couplings among relevant component processes; couplings that reduce the degrees of freedom among the possible arrangements of the components of mind and body to produce speech (Tuller et al., 1982; Turvey, 1990, 2007). Synergies impose constraints that compress the potential degrees of freedom in the possibilities for articulation or action.

A mechanical example of a coordinative link is a tie-rod that connects the two front wheels of a vehicle. Two degrees of freedom are required to independently control each wheel. But any wheel arrangement that points them in different directions is dysfunctional. A tie-rod coordinates their movements and reduces the control problem to a single degree of freedom, instantiated in the position of the steering wheel. Of course biological coordinative synergies are more subtle, flexible, and abstract, but the essentials of the solution are the same (Tuller et al., 1982; Riley et al., 2011).

Kelso et al. (1984) conducted a seminal test of the synergy hypothesis in speech production. The task required each participant to say “bab” and “baz” despite an unpredictable, occasional, mid-utterance, force perturbation to their jaw. The applied force altered the participant’s articulatory trajectory. In the case of “bab,” participants’ upper and lower lip movements immediately compensated for the jaw perturbation such that no distortion in the utterance could be detected (<30 ms, arguably faster than the minimum lag of a sensory-motor loop). Similarly, the same jaw perturbation while saying “baz” yielded virtually instantaneous and appropriate adaptation in the tongue’s trajectory that again left no distortion in the final utterance. The linkages that compose synergies predict such online, reciprocal compensation among the components of speech articulation.

What is most remarkable is the local, online compensation that emerges to render the intended utterance fully intact. The linkages that compose the synergies allow simultaneous reciprocal compensation among the elements of the pronunciation of “bab” and “baz.” Speech acts, such as word pronunciation, are accomplished through coordinative synergies (Kelso et al., 1984, 1986; Saltzman et al., 1998; Turvey, 2007; for a review, see Van Lieshout, 2004). Even elementary speech gestures require cooperative relationships among ensembles of elemental perceptual, cognitive, and neuromuscular variables. The online, reciprocal compensation that is observed at the behavioral level is an expression of *interaction dominant dynamics* (Jensen, 1998; Van Orden et al., 2003a, 2005; Kello et al., 2008;Holden et al., 2009, 2011). This category of coordinative behavior is also indicative of biological self-organization in general, and represents an established instance of self-organization in speaking aloud.

### Linking synergies to a distribution’s shape

We now explain how measures of pronunciation time are fundamentally related to the coordinative synergies that are known to govern natural speech production. Pronunciation time distributions offer a probabilistic event-based assessment of the coordinative dynamics that arise to support performance for each target item, or so we claim. Pronunciation times, themselves, measure the time required for an utterance to coordinate and unfold to a point where a participant’s audible voice-amplitude exceeds a threshold of measurement.

While this coordinative activity unfolds over time, it is nevertheless sampled as a discrete “pronunciation time” once an utterance triggers a voice key. As such, pronunciation times supply point-samples of the evolving multidimensional dynamics of physiological, anatomical, and acoustic variables as they unfold and give rise to a spoken word. Instantaneous pronunciation time measures are thus substituted for an underlying, continuous dynamic trajectory and that trajectory is formally collapsed into a point process (Lowen and Teich, 2005). Clearly, some portion of each pronunciation time is unique to the particulars of the target word: Its unique acoustic profile, measurement uncertainty, and other idiosyncrasies. However, all aspects of the dynamic must enfold to yield a successful, audible word pronunciation.

It is well known that cognitive, kinematic, and articulatory manipulations routinely influence *mean* pronunciation and response time. This fact establishes that both pronunciation and response time measurements are sensitive to a broad spectrum of performance dynamics (e.g., Abrams and Balota, 1991; Van Orden and Goldinger, 1994; Van Orden et al., 2003b; Perry et al., 2010). In fact, the discovery that coordinative synergies support speech performance implicates speech as dynamically assembled and governed. If so, dynamical systems theory dictates that the trajectories entailed by the larger “speaking-a-printed-word” system are in evidence in the pronunciation time measurements. This is key consequence of the interconnectedness of dynamical systems.

Events that affect the dynamics of one process reverberate through and change the dynamics of other process because they are reciprocally intertwined. This coupling across degrees of freedom means that appropriate measurements of just a *single* observable normally reveals information about the dynamics of the *system as a whole* (e.g., Takens, 1981). In fact, Takens’ embedding theorem and related findings gave rise to an entire signal-processing discipline dedicated to uncovering and describing the dynamics of complex systems based, in many cases, on just a *single* observable (Abarbanel, 1995; Kantz and Schreiber, 1997; Gao et al., 2007). Embedding theorems are as foundational to the statistics of non-linear dynamic systems as the Central Limit theorem is to the statistics of stochastic linear systems.

Our working hypothesis is that if coordinative synergies underlie acts of reading and speaking words aloud, it will be corroborated in the shape of pronunciation distributions. There are two ideal probability distributions that are symptomatic of discrete samples of the aforementioned mutually contingent, coupled dynamics (Montroll and Shlesinger, 1982; West and Deering, 1995; Holden et al., 2009). The first is a *lognormal distribution* (Evans et al., 2000; Limpert et al., 2001). It is a positively skewed distribution that appears as a symmetric Gaussian distribution after a logarithmic transform of the measured variable. The second, an *inverse power law* distribution, expresses a more pronounced positive skew than a lognormal distribution (Clauset et al., 2009). Power law behavior is symptomatic of self-organizing physical systems poised are near a critical point (Bak, 1996; Jensen, 1998).

### Article overview

Relating standard pronunciation time measurements to an underlying, largely unobserved dynamic flow requires a basis in evidence. It must be demonstrated that pronunciation times are plausibly associated with interaction dominant dynamics. The detailed analyses of this article are directed at establishing whether pronunciation time distributions conform to shapes that are consistent with event-based samples of coordinated dynamic flows. Thus, we seek evidence of interaction dominant dynamics from the pronunciation time distributions of individual participants. If pronunciation time distributions reasonably conform to either idealized lognormal, inverse power law, or mixtures of these distributions then pronunciation events are likely contingent on interaction dominant dynamics. After all, both classes of distributions are widely identified as symptomatic of interaction dominant dynamical systems in nature (Montroll and Shlesinger, 1982; West and Deering, 1995; Bak, 1996; Jensen, 1998; Limpert et al., 2001; Holden et al., 2009).

Over the course of this article, a refined incarnation of the Holden et al. (2009) cocktail description based on mixtures of lognormal and inverse power law distributions is introduced. Its key advantage is its suitability for maximum-likelihood fitting techniques. The refined description facilitates density estimation and hazard function tests that contrast common exponential and inverse power law descriptions of individual participant’s pronunciation time distributions. The outcomes of our tests indicate that power law behavior is likely a more accurate description of the stretched, slow tails of individual participant’s pronunciation time distributions than exponential behavior.

So far, we have detailed how pronunciation times, traditionally viewed as indexes of processing time, can be reinterpreted as indicators of the dynamic coupling entailed in the act of speaking printed words. In the next section, we introduce and contrast distributions that are symptomatic of superposed, component dominant dynamics, such as an exponential or a Gaussian, from those symptomatic of interaction dominant dynamics, such as an inverse power law or a lognormal distribution. The correspondence test section details the findings of our exponential versus power law statistical contrasts on a 30 participant pronunciation time data set from Experiment 2 of Holden et al. (2009). Following that, the same contrasts are generalized to a much larger 470 participant pronunciation time data set from the English Lexicon Project (ELP) described by Balota et al. (2007). In both cases, mixtures of lognormal and inverse power law distributions described the empirical patterns reliably better than ex-Gaussian or ex-Wald alternatives. Finally, our general discussion illustrates how interaction dominant dynamics might impact the interpretation of several standard empirical word recognition results. We illustrate how the understanding of linear item-level regression analyses on large-scale pronunciation time data sets must be conditioned by the fact the cognitive dynamics word recognition tend to unfold on faster times-scales than articulatory dynamics.

## Distinguishing Exponential from Power Law Behavior

In science, some distinctions matter more than others. Critical differences involve the identification of patterns with broad implications for the core assumptions of a research program. For cognitive scientists, determining whether exponential decay or inverse power law decay best characterizes the slow tails of pronunciation time distributions is just such a crucial question – the answer could at once render large classes of cognitive models as either plausible or implausible.

### Exponential density functions

Historically the positive skew commonly expressed in both pronunciation and standard response time distributions is approximated as a form of exponential decay. That is, past the distribution’s mode the probability density is thought to decay as *p*(*t*) ≈ (1/λ)e^−t^ where *t* is just the time axis of pronunciation time. Exponential decay is, for instance, the dominant term in the slow tails of the ex-Gaussian, the ex-Wald, the Gamma, and the inverse Gaussian distributions (Moscoso Del Prado Martín, 2010). Each distribution has been put forward as either a description or a model of response time distributions (e.g., see Luce, 1986; Van Zandt, 2000; Schwarz, 2001). In all that follows, we adopt the ex-Gaussian and the ex-Wald distributions as canonical examples of pronunciation time descriptions that express exponential decay.

An exponential tail could signify, for example, cognitive and perceptual processes that conform to stochastic “counting” or queuing output processes: The steady, reliable accrual of cognitive or perceptual information, as characterized by the mean (λ) of the distribution (e.g., Townsend and Ashby, 1983; Balakrishnan and Ashby, 1991). Furthermore, if an exponential rate parameter is sufficient to characterize a cognitive process then it could, in principle, be identified and discriminated from other processes with different characteristic rate parameters or distribution functions. Finding in favor of an exponential description of the slow tails of pronunciation time distributions supports the hypothesis that cognitive components, themselves, dominate the transactions associated with reading and speech performance – a finite set of independent, superposed processes that conform to a characteristic time-scale – indicated by a set of characteristic exponential rate parameters, for instance. Given this outcome, the reductive methods of linear analysis may best reveal and individuate the fundamental processes that govern reading, speaking, and related cognitive activities.

#### The ex-Gaussian description

The ex-Gaussian distribution represents the convolution of a Gaussian distribution and an exponential distribution. The convolution operation yields a “child” random variable that represents the sum of these two independent “parent” random variables. The original description of the ex-Gaussian was rooted in a hypothesis that response times represented the serial output of two processing stages, a perceptual-decision stage that yields an exponential variate and a motor-execution stage that results in a Gaussian variate (Hohle, 1965). An opposing interpretation was also proposed, where the motor response generated an exponential and the perceptual-decision stage was Gaussian (McGill and Gibbon, 1965). Some contemporary authors limit their use of the ex-Gaussian to a purely descriptive role (e.g., Heathcote et al., 1991; Balota and Yap, 2011; Yap et al., 2012) while others have interpreted the ex-Gaussian parameters in terms of distinct cognitive process (e.g., Balota and Spieler, 1999). Still others question the motivation for using the ex-Gaussian at all, given that it lacks the peaked hazard function that is widely indicated in empirical response time distributions (e.g., Schwarz, 2001; Holden et al., 2009; Van Zandt, unpublished manuscript).

#### The ex-Wald description

The ex-Wald distribution represents an additive combination of a Wald (a.k.a., an inverse Gaussian) and an exponential distribution (Schwarz, 2001). It is aimed at describing cognitive processes that entail an additive combination of a bottom-up accumulation of information that outputs variation in the form of a Wald distribution, plus a non-decision process associated with a response threshold that yields exponentially distributed variability. The resulting convolution of a Wald and an exponential results in an ex-Wald distribution.

The ex-Wald distribution is typically implemented as an additive, three parameter convolution of the parent Wald and exponential distributions which facilitates selective influence tests (Schwarz, 2001). We implemented a more flexible *four-*parameter version of the ex-Wald that includes an onset threshold for the exponential portion of the distribution. The added parameter allows the model to capture a broader potential set of functional forms than its three parameter counterpart. Since the ex-Gaussian uses three parameters, we added a fourth parameter to the ex-Wald to insure that a lack of flexibility resulting from too few parameters could not be the sole reason that an exponential description could succeed or fail to capture the empirical patterns.

### Inverse power law density functions

More recently cognitive scientists introduced descriptions of pronunciation and response time distributions that posit inverse power law decay as the dominant term describing the positive skew in the slow tails of response time distributions. If the extreme slow tail of a distribution decays as a power function, then the probability of observing a particular response time, *p*(*t*), is the inverse of the pronunciation time value, *t*, itself, raised to a scaling exponent α, i.e., *p*(*t*) ≈ *t^-α^*. An inverse power law entails a more dramatic positive skew than an exponential. Examples include the Pareto distribution, Fieller’s distribution (Moscoso Del Prado Martín, 2010), the Lévy distribution (Rhodes and Turvey, 2007), power law tail behavior (Sigman et al., 2010), and the cocktail description (Holden et al., 2009). We adopt a refined version of the Holden et al. cocktail description as a canonical example of pronunciation and response time models that express power law behavior in their slow tails.

An inverse power law tail implies the processes supporting reading and speaking aloud are capable of spanning a wide range of time scales, from very short to very long. Power law behavior is associated with complex systems composed of processes that interact to self-organize their behavior across multiple temporal or spatial scales (Jensen, 1998; Van Orden et al., 2003a). The behavior of countless complex systems relies on strongly coupled, interdependent processes that yield emergent patterns, such as power law distributions (Bak, 1996; Johnson, 2007). If power law behavior is commonly expressed in naming performance then the tools of complexity science may best aid investigations concerning the fundamental principles and patterns expressed in speaking printed words aloud.

The catch, however, is that in practice exponential and power law functions, such as probability densities, are notoriously difficult distinguish empirically since both functions are so similar (Clauset et al., 2009). In the context of favorable parameter settings each function can be made to closely mimic the other. Our statistical analyses are aimed at making just such a distinction regarding the tail behavior of pronunciation time distributions derived from the speeded word naming task. As we explain, the hazard functions of the exponential and power law distributions are quite distinct, despite the similarity of their density functions. Thus, a distinction between exponential and power law decay in the slow tails of a distribution does have the potential to be determined.

### Pronunciation times as mixtures of lognormal and power law samples

Each naming trial requires participants to pronounce aloud an individual word that appears on a visual display. The resulting pronunciation time, the elapsed time between the presentation of the word and the moment a participant begins to utter the word, is then recorded. Normally, participants are asked to name hundreds of words in a single session, or perhaps thousands of words across several sessions.

**Figure 1:**
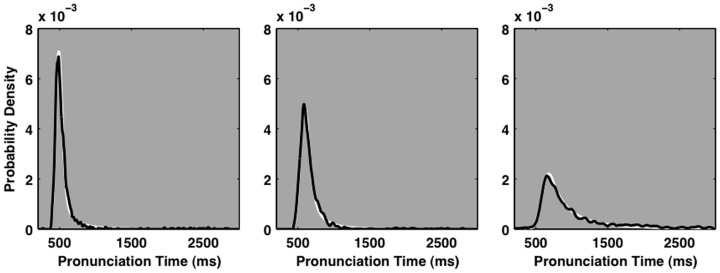
**Three example empirical pronunciation time distributions from the English Lexicon Project data set (Balota et al., 2007)**. Each plot depicts the probability density function of an individual participant’s empirical pronunciation time distribution. The black lines represent kernel-smoothed empirical probability density functions. Maximum-likelihood fits of the refined cocktail description are depicted in white, behind the empirical density functions. In each plot, the white line depicting the cocktail fit is hardly visible because it closely matches the empirical distribution.

Individual pronunciation times produced by different individuals are heterogeneously distributed. Figure 1 allows a cursory contrast of three individual ELP participant’s pronunciation time distributions. It reveals an apparently broad continuum of probability density functions. The distribution in the left plot is compact and nearly symmetric, the distribution in the right plot reveals a clear and potent positive skew, and the shape of the distribution in the center plot falls between these two extremes. In lay terms, some participants are just faster more often than others. However the differences in the shapes of the distributions across participants are striking. Arguably, any accurate characterization of the variety of shapes of pronunciation time distributions is a critical first step in providing a basis for understanding the process that governs the act of speaking aloud a printed word. This and other concerns motivated the so-called cocktail model. It mixes samples from two idealized distributions, proportionally, like the molecules of different liquids in a cocktail.

#### The refined cocktail description

The original Holden et al. (2009) cocktail description approximated empirical pronunciation time distributions as a probabilistic mixture of synthetic “trial” samples from either a lognormal or an inverse power law distribution. The refined cocktail model is a simpler and more compact parametric formulation of the original lognormal and inverse power law mixture distributions. The lognormal mean and standard deviation are treated as unknown free parameters. The inverse power law density entails two unknown free parameters including an onset threshold (a necessarily positive-valued lower bound of support for the distribution) and a scaling exponent.

The refined model is formulated such that it is a pure lognormal distribution for the values of the random variable below the power law threshold (the “front” or left side of the distribution). Above the threshold (the “tail” or right side of the distribution), the refined cocktail is a mixture of a lognormal and an inverse power law distribution with unknown weights. The refined cocktail model is defined so that it is guaranteed to be a continuous and smooth probability density function over the entire positive real line – except at the threshold value – depending on the weights of the mixture components the density may become discontinuous at the threshold. Thus, an interrelationship or constraint among the parameters of the cocktail model components and their mixture weights was imposed to ensure an idealized *smooth*, *unimodal* probability density function, that by definition, integrates to unit area.

The refined cocktail model is a function of four independent parameters: the lognormal distribution mean and standard deviation (Ω_LN_ and σ), the inverse power law distribution scaling exponent (α), and the relative weight of the power law tail in its mixture with the portion of the lognormal that falls above the lognormal mean (ρ_PL_). Three remaining parameters are all implicitly dependent on the other four free parameters (see Appendix). For a given pronunciation time dataset, the cocktail model parameters can be estimated with standard parameter estimation techniques, such as maximum-likelihood estimation or maximum spacing estimation. The crux motivation for the cocktail mixture is the hypothesis that the dynamics of word naming vary largely in terms of their relative stability, and span a continuum ranging from relatively stable lognormal behavior to less stable inverse power law behavior.

## A Correspondence Test of the Original and Refined Cocktail Models

There are any number of ways one could implement a probabilistic mixture of lognormal and power law samples. Thus, it is important that the results of the refined cocktail mixture display evidence of correspondence to the original cocktail description. The correspondence test amounted to comparing the parameters from the original and refined cocktail descriptions to fits of the pronunciation time data from Experiment 2 of Holden et al. (2009).

We also used the ex-Gaussian and ex-Wald models to approximate the same empirical distributions. Parameter estimates from all three models were then used to introduce and apply a rigorous goodness of fit test recommended by Clauset et al. (2009) for distinguishing heavy-tailed distributions, such as the power law and exponential. The test is based on a Monte Carlo version of the standard Kolmogorov–Smirnov goodness of fit statistic. Following that, we introduce a novel hazard function analysis, using the Bayesian Information Criterion or BIC statistic, that contrasts mean integrated squared error between the empirical and ideal best-fit cocktail, ex-Gaussian, and ex-Wald hazard functions.

Since the experimental procedures used to collect these data are described elsewhere, the bulk of our Method section supplies the details and rationale of our statistical procedures. Our Results and Discussion sections offer brief overviews of each of these procedures, however. The full derivation of the refined cocktail model appears in an Appendix. Matlab^®^ code is available at http://homepages.uc.edu/~holdenjn/. Readers that do not require all the details of our statistical procedures may choose to skip the Method section of this experiment.

### Method

#### Participants and procedure

The Holden et al. (2009) Experiment 2 data set is composed of 30 participants’ pronunciation times to 1100 randomly selected single and multisyllabic English words that ranged from 4 to 15 letters in length with an average frequency of occurrence of 70.2 times per million (SD = 295.16) according to the Kuĉera and Francis (1967) norms. Readers may refer to the original article for a more complete description of the word stimuli and laboratory methods.

#### Distribution fitting

Standard maximum-likelihood estimation methods were applied to the empirical distributions to estimate a set of density function parameters for each participant’s pronunciation time distribution. Each participants’ distribution was approximated using the refined cocktail distribution, the ex-Wald distribution, and the ex-Gaussian distribution.

One typically uses a Kolmogorov–Smirnov (K–S) goodness of fit test to determine the relative match between an empirical distribution and a candidate model distribution. The test returns the maximum distance (*D*) between the two distributions over the interval of their cumulative density functions. If *D* is small enough, there is *insufficient* evidence to reject the model as a description of the data.

However, the assumptions of the above testing scheme are valid only if the model is known *a priori* – and that criterion is so far unmet for pronunciation times. If the *D* value is derived from a contrast between the best-fit model and the empirical distribution, then a correlation between the data and model arises. Thus, we used a more rigorous Monte Carlo procedure to generate an unbiased goodness of fit test (Clauset et al., 2009). First, for each model, and each participant, an ideal best-fit cumulative distribution function was inverted to generate simulated pronunciation times of the same size as the participant’s empirical distribution. For a given model, 2500 synthetic data sets were generated using the best-fit model parameters. Next, each synthetic distribution was compared to the idealized model distribution function, using the same parameters, and a *D* goodness of fit statistic was generated for each synthetic data set. This operation yielded a distribution of 2500 *D* values resulting from the synthetic fits. Finally, we compared the *D* statistic derived from the given model’s best-fit of the participant’s empirical data set to the distribution of 2500 *D* values derived from all the synthetic fits.

Goodness of fit was defined in the following manner: If the *observed D* statistic fell on or *below* the 90th percentile (i.e., *p* ≥ 0.1) of the distribution of *synthetic D* statistics, the model *could not* be reasonably ruled out as a plausible description of the empirical data. In our Section Results, we report the number and percent of empirical distributions that passed this test. Next, we describe an additional test, aimed at contrasting the hazard functions of each model.

#### Hazard function estimation

We estimated empirical hazard functions by first computing a variable-width Gaussian-kernel probability density and cumulative distribution estimates for each individual’s pronunciation time distribution (Silverman, 1986). At each point on the increasing *X*-axis of pronunciation time *t*, the probability density is *f*(*t*) and the cumulative distribution function is *F*(*t*). The empirical hazard function, *h*(*t*) was computed as *h*(*t*) = *f*(*t*)/[1 − *F*(*t*)]. Likewise, ideal model hazard functions were computed on the density and cumulative distribution functions specified by the maximum-likelihood fits of each participant’s empirical distribution using the cocktail, ex-Wald, and ex-Gaussian distributions. Hazard functions were computed over 1024 equally spaced points on the interval, beginning with the minimum pronunciation time censoring value and ending with the largest *observed* pronunciation time in each participant’s empirical distribution. The empirical and ideal hazard functions were computed over the same pronunciation time intervals for each participant. We contrasted empirical and model hazard functions by first computing the mean squared integrated error (MISE) between them (i.e., MISE, Silverman). We compared the three models with a BIC statistic (i.e., BIC, e.g., Schwarz, 1978; Wagenmakers and Farrell, 2004; Chatterjee and Hadi, 2006) was computed as:

(1) $$\text{BIC}=n\times \ln\left(\frac{\text{MISE}}{n-1}\right)+\text{θ}\times \left(\ln n\right)$$

where *n* is the number of pronunciation times in the empirical distribution, MISE is the mean integrated squared error, θ is the number of free parameters in the model (three for the ex-Gaussian, four for the cocktail and ex-Wald). In this form, lower BIC values indicate relatively better fits than larger values. The model with the lowest BIC score was classified as the winning description.

We also address a need for an alternative and more flexible hazard function routine than the classic Miller and Singpurwalla (1980) method (see Discussion). We implemented a routine based on a Gaussian-kernel-smoothed probability density estimate that used a variable-width kernel. The empirical kernel-smoothed density functions were transformed into hazard functions and used in contrasts with ideal hazard functions based on the best-fit model for each candidate model. The main advantage of using the Gaussian-kernel method lies in its ability to represent a wider range of hazard function shapes than the randomized smoothing methods that were the historical standard in response time analyses.

The hazard contrast represents a separate, quasi-independent statistical test from the maximum-likelihood fits of the models. On one hand, it depends on the fits, on the other hand, the model and empirical hazard functions are generated and compared by a wholly different means. We adopted procedures to guard against biasing the analyses in favor of either the exponential or power law hypothesis. First, the asymptotic tail behavior of hazard functions computed in the manner described is to increase toward positive infinity as the denominator [1 − *F*(*t*)] approaches zero, but an exponential hazard is constant and a power law hazard decays toward zero. Second, we applied the BIC statistic to compensate for the fact that both the cocktail and ex-Wald entail one more free parameter than the ex-Gaussian distribution. Given a distribution with 1100 observations the BIC penalty for the ex-Gaussian distribution is 21.00 and 28.01 for the more flexible cocktail and ex-Wald models which represents a 25% increase in the penalty score for one additional free parameter. Next we describe, in turn, the outcome of the maximum-likelihood fitting and hazard discrimination analyses.

### Results

#### Distribution fitting outcomes

All statistical analyses included both correct and incorrect pronunciation times that fell between 350 and 3500 ms. The small proportion of error pronunciation times (2.45%) were included because they made no difference in the outcome of our analyses. Twenty-seven of the 30 participants distribution’s were reasonably approximated by the refined cocktail model (90%). Using the same Monte Carlo standard for goodness of fit described in the methods, the ex-Gaussian plausibly described 57% of the data sets. The Monte Carlo test for goodness of fit revealed that the four-parameter ex-Wald distribution plausibly described only 50% of the data sets. Our procedure for assessing goodness of fit was described in the Method section.

At the level of the density function, the cocktail model successfully approximated the individual participant’s pronunciation time distributions within the confines of the rigorous statistical standards recommended by Clauset et al. (2009). The ex-Gaussian captured every distribution that was successfully approximated by the ex-Wald. Neither the larger ex-Wald parameter set, nor the lack of a penalty for more parameters enhanced its ability to capture empirical patterns at the level of the density function.

The fitting outcome is informative on several points. First, the cocktail distribution successfully approximated a much larger portion of the empirical distributions than either the ex-Gaussian or ex-Wald models. The success of the cocktail distribution is not likely due exclusively to the fact that it has more free parameters than the ex-Gaussian. The ex-Wald entailed the same number of free parameters as the cocktail distribution, but it actually fared worse than even the three parameter ex-Gaussian. It therefore does not appear that we are exclusively observing the impact of fitting more or less flexible candidate descriptions of the pronunciation time distributions.

#### Cocktail correspondence assessment

The key location parameters estimated using the original and refined versions of the cocktail model agreed with each other surprisingly well. The average values of the four free refined cocktail parameters were: lognormal mean Ω_LN_, 6.31, or 550 ms, (SD = 0.12), lognormal SD, σ, 0.12 (SD = 0.01), scaling exponent, α, 6.68 (SD = 1), and the power law proportion tail, ρ_PL_, 0.33 (SD = 0.16). Among the 26 distributions that were plausibly approximated by both the original and refined cocktail, the lognormal mean (Ω_LN_), the power law scaling exponent (α), power law threshold (Ω_PL_) were strongly correlated with the same parameters derived from the original cocktail description *r*(24) = 0.97, *p* < 0.05 and *r*(24) = 0.91, *p* < 0.05 and *r*(26) = 0.93, *p* < 0.05 respectively. Regression analyses revealed slopes very near 1 and intercepts near 0 for these three parameters.

The free parameters that correspond to variability, such as the lognormal SD (σ) and the power law weight parameter (ρ_PL_) were also positively correlated, but less so, *r*(24) = 0.53, *p* < 0.05 and *r*(24) = 0.65, *p* < 0.05 for σ and ρ_PL_, respectively. The primary difference between the two descriptions was that the refined cocktail model successfully approximates the tails of the more heavy-tailed empirical distribution’s with a lower weight or proportion of samples from a lognormal distribution in the distribution’s right tail. The main discrepancy between the two descriptions loads on variables that describe variability because the original cocktail fits were derived from contrasts of synthetic and empirical kernel density functions, both of which relied on automatic smoothing parameters that increase relative variability as the square of the smoothing parameter (Silverman, 1986, p. 37). Overall the correspondence between the original and refined implementations of the cocktail description is quite remarkable given they were arrived at by such disparate means.

Next we present an additional evaluation, focused on describing the tail behavior of the distributions. It is based on hazard function contrasts. Both the ex-Gaussian and the ex-Wald descriptions of pronunciation time entail exponential tails but the cocktail model entails power law decay. Transforming the density and distribution functions into the hazard function domain amplifies their differences and often renders a categorical distinction between exponential and power law tail behavior.

#### Hazard function contrasts

Mathematical psychologists articulated several empirical criteria that any general description of response time distributions should approximate. The consensus regarding the empirical standards that must be demonstrated, in order of relative difficulty, are: (1) capture the descriptive statistics of response times, e.g., mean, SD, and skew. (2) Recover the cumulative distribution and probability density functions of response time. (3) Express the three characteristic hazard functions of response time (e.g., see Luce, 1986; Townsend, 1990). To avoid the pitfalls of *ad hoc* statistical mimicking, all this must be accomplished in the context of a compelling theoretical motivation that arises for reasons beyond the observed empirical patterns (Van Zandt and Ratcliff, 1995).

**Figure 2:**
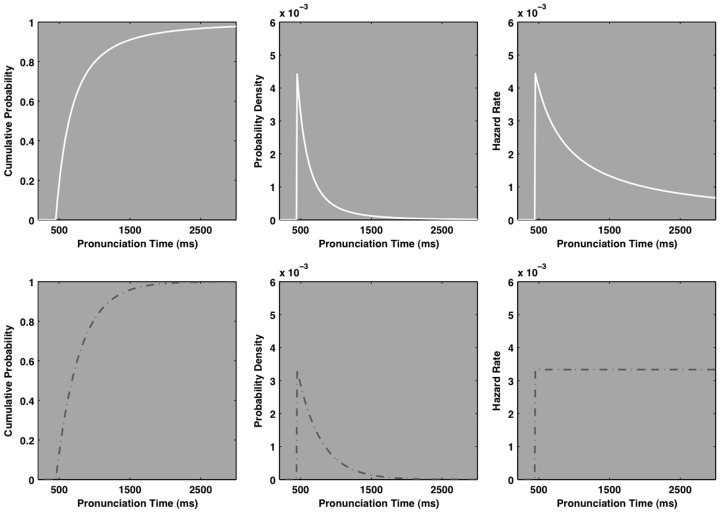
**The ideal cumulative distribution, probability density, and hazard functions of a pure power law distribution (solid white line, top three plots), and a pure exponential distribution (dashed-gray line, bottom three plots)**. The cumulative distribution and probability density functions of each distribution are visually quite similar, but the hazard functions are qualitatively distinct. The power law hazard, itself, decays as a power law. By contrast, the exponential hazard is constant. The BIC mean squared integrated error analysis capitalizes on the qualitative and quantitative difference in the hazard domain.

A probability density function depicts the instantaneous probability of observing an event within an interval on the *X*-axis. Similarly, a hazard function describes an instantaneous event rate, per unit time, *given that it has not yet occurred*. Hazard functions tend to amplify subtle quantitative differences among distributions so they become visible, qualitative differences. Figure 2 illustrates that while the probability density functions of the exponential and power law distributions are reasonably similar, their hazard functions are quite distinct.

An exponential distribution’s hazard function is a constant function of time. That is, it rises to a constant, asymptotic rate as depicted in bottom right plot of Figure 2. This fact is the basis of the characterization of an exponential process as “memory less.” The instantaneous event rate, given that an event has not yet occurred, is constant. That is, the ratio between elapsed events and potential remaining events is constant for any given interval of time. Hence the saying “used is as good as new” is directed at tools and machinery that break or otherwise fail as an exponential function of time. For a given interval of time, a brand new tool is just as likely to fail as a very old tool, so there is no benefit to paying the typical premium for “newness.”

The ex-Gaussian distribution yields a hazard function that rises monotonically to a constant asymptote. This behavior straightforwardly reflects its constituent Gaussian and exponential ingredients. A Gaussian distribution’s hazard function is increasing and the exponential distribution’s hazard function is constant.

The ex-Wald hazard function’s behavior is more complex. It can display a peaked then decaying hazard that resembles a lognormal hazard function. The basis of its peak is the Wald distribution, which itself has a peaked hazard function. In the context of certain parameters, the ex-Wald hazard function can (1) rise above an ex-Gaussian fit to the same data (2) closely mimic the increasing then constant ex-Gaussian hazard function, and (3) display a peaked and decreasing hazard function that resembles that of the cocktail model. This is the basic pattern that must be expressed to capture typical empirical pronunciation time hazard functions. Thus the superposition of a Wald distribution and a variable threshold exponential tail allows the ex-Wald to exhibit the hazard behavior of both its constituents. However, the normal tendency of the ex-Wald hazard is to maintain higher overall hazard rates than a comparable cocktail hazard function, due to its basis in exponential rather than power law decay.

Lognormal and inverse power law distributions both yield *peaked* hazard functions, they rise quickly to a maximum and then decay toward zero past that point. The peak and rate of decay of the lognormal hazard function is governed largely by the standard deviation of the distribution. The ideal hazard function of an inverse power law distribution itself decays as a power law with a scaling exponent of 1, regardless of the scaling exponent of the distribution’s probability density function, as in the top right plot of Figure 2. Thus, an ideal power law hazard function, itself, is scale-free, as is the power law density function. While specific details of the hazard functions depend on the distribution’s parameter values, the cocktail model often yields peaked hazard functions that decay more rapidly than the exponentially based hazard functions.

Hazard functions are normally assessed qualitatively, for peaks and other asymptotic behavior. This is due in part to a lack well-established quantitative methods that can be used to distinguish hazard functions. Our Method section describes the details of a test we developed to contrast the hazard functions of the three models. It adopts a BIC statistic to evaluate the MISE between the empirical hazard function and each best-fit model’s hazard function (Silverman, 1986). According to the BIC hazard test the cocktail hazard was a better description of 24 of the 30 (80%) empirical hazard functions. The ex-Gaussian and ex-Wald each successfully captured 3 of the 30 hazard functions for an exponential total of six (20%) of the empirical distributions.

**Figure 3:**
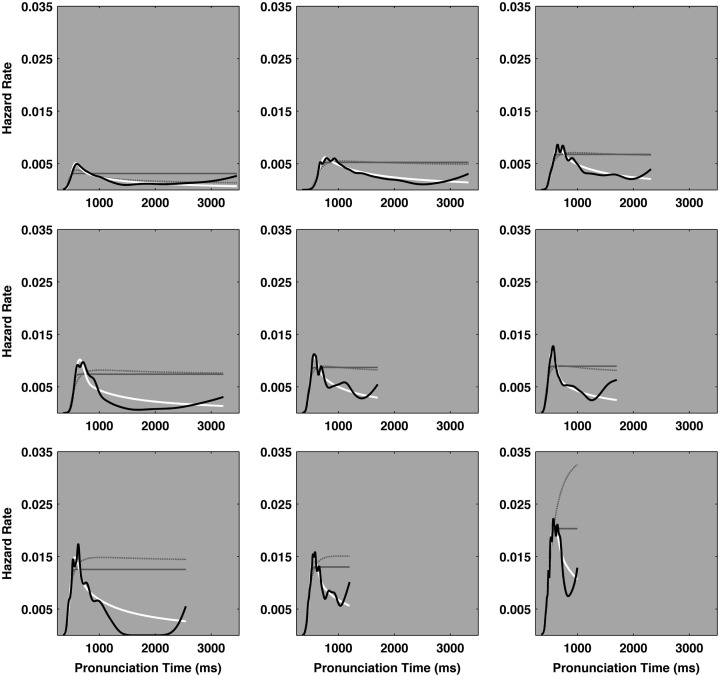
**The *X*-axes of each plot track pronunciation time and the *Y*-axes indicate the hazard rate, in events per millisecond**. The heavy solid black line represents the empirical hazard functions for each of the nine participants. The ideal hazard functions for cocktail description are depicted as heavy white lines. The hazard functions of the ex-Gaussian and ex-Wald models are depicted as lighter, solid gray, and dashed-gray lines, respectively. Cases that appear to only depict one solid gray line indicate the ex-Gaussian and ex-Wald hazard functions were effectively identical. The majority of the empirical hazard functions entail a peak and their slow tails more closely match the power law decay indicated in the cocktail description than the exponential decay indicated by either the ex-Gaussian or ex-Wald alternatives. The distribution in the top left plot yielded the smallest power law scaling exponent (α = 3.44), the bottom right plot yielded the largest scaling exponent (α = 11.36). α = power law scaling exponent.

Figure 3 illustrates the empirical and the three ideal hazard functions for each of the nine participants in the experiment. In each plot, the solid black line represents an individual participant’s empirical pronunciation time hazard function. The solid white line depicts the ideal cocktail hazard function corresponding to the best-fit parameters, as estimated from the empirical distribution. The lighter solid and dashed-gray lines depict the hazard functions of the best-fit ex-Gaussian and ex-Wald distributions, respectively. In four cases both exponential hazard functions fell nearly atop one another, which resembles a single solid gray line on the plots. The hazard functions of the leading edges of all the model distributions were highly similar. However the hazard functions of the exponential and power law descriptions tended to diverge in the slow tails of the model distributions. Overall, the pattern of decay in the tails of the empirical distributions was better represented by the power law behavior entailed in the cocktail description than by the exponential behavior entailed in the ex-Gaussian and ex-Wald descriptions.

#### Benchmarking simulations

We assessed our BIC hazard discrimination routine by replacing, in turn, each of the 30 participant’s empirical pronunciation times with *synthetic* data derived from the best-fit parameters of the cocktail, the ex-Gaussian, and ex-Wald models (also, see Wagenmakers et al., 2004 for a similar technique). Replacing the empirical data with data from known models allowed us to assess the ability of the hazard routine to discriminate the power law from exponential models. Twenty-five realizations of the 30 participant simulations were run for each ideal model. Correct classification (hit) rates and incorrect classification (false alarm) rates were averaged across the 25 realizations of each 30 participant simulation.

The winning model was that model with the lowest BIC score for the given simulated data, as specified by the parameters of one of the three models. If either the ex-Gaussian or ex-Wald model were the true models, we treated a win for either model as a win for an exponential description. The ex-Gaussian description often captured ex-Wald data since they are functionally similar and the ex-Gaussian entails one less parameter. Similarly, if the cocktail description was the true model, we treated a win for either the ex-Gaussian or ex-Wald as an exponential false alarm.

When the cocktail model was the true model, it was correctly classified as a power law 89% of the time, the exponential false alarm rate was 11%. Given an ex-Gaussian as a true model, it was accurately classified as exponential 90% of the time, with a power law false alarm rate of 10%. A true ex-Wald was classified as exponential 98% of the time, with a power law false alarm rate of only 2%. These hit and false alarm rates, in turn, allowed us to estimate the sensitivity of the hazard classification routine with the help of a *d*-prime (*d*′) analysis.

The ability of the BIC hazard classification routine to distinguish the expression of exponential and power law behavior, its sensitivity, was estimated by computing a *d*′ statistic for each hit and false alarm rate. The *d*′ computation works by transforming hit and false alarm rates into a standardized *z*-score that represents the distance between the means of two hypothetical Gaussian distributions that represent the “signal” (true model) and “noise” (alternate model) distributions (Green and Swets, 1988). The values of *d*′ for the power law versus exponential contrasts using synthetic data were 3.95, 2.56, and 2.49 for the ex-Wald, the ex-Gaussian, and the cocktail description, respectively.

So, given the ranges of parameters represented by this data set, the ex-Wald was the easiest to classify as an exponential model. This was despite the ex-Wald being the most flexible exponential model, and likely resulted from the BIC penalty imposed for the additional parameter. The ex-Gaussian was a bit more difficult to distinguish from the power law, as it was occasionally misclassified as such. Likewise, the cocktail model was occasionally misclassified as an exponential. The fact that the cocktail description and the ex-Gaussian yielded comparable *d*′ values indicates that, on average, the BIC adjustment produced a reasonably balanced contrast between the simplest exponential model the more complex cocktail description.

### Discussion

The maximum-likelihood fits to the three ideal models strongly favored the cocktail description of the pronunciation time distributions over an ex-Gaussian or a four-parameter version of the ex-Wald distribution. Our density fitting methods were conservative in that we followed Clauset et al.’s (2009) criteria for evaluating the plausibility of each model. The technique was explicitly developed to distinguish different classes of heavy-tailed distributions, such as the exponential and power law. Ninety percent (90%) of the participant’s distributions could be plausibly approximated using the cocktail description. By contrast the ex-Gaussian plausibly approximated only 57% of the distributions and the ex-Wald passed muster only 50% of the time.

The BIC hazard classification routine took model complexity explicitly into account by comparing goodness of fit with a BIC statistic that strongly punishes more complex models over simpler models (Wagenmakers and Farrell, 2004). This test specifically examined the hazard function shapes. The hazard shape is a highly diagnostic property, not readily represented in outcomes of goodness of fit tests conducted on density functions. The BIC hazard test classified 80% of the empirical distributions as most plausibly consistent with the cocktail description of pronunciation times, the two exponential models captured the remaining 20% of the distributions.

#### Alternate censorship criteria

Notably, imposing a common but more liberal response trimming criteria from the ex-Gaussian literature allowed the ex-Gaussian to successfully fit the density functions of 29 of the 30 participant’s distributions (by contrast, the cocktail captured 25 of 30, the ex-Wald, 19 of 30). This censoring method removes errors, observations beyond 200 and 3000 ms as well as any observations beyond 2.5 standard deviations from the distribution’s mean. Consequently, the ex-Gaussian captured five additional distributions that it otherwise failed to fit; all of which previously appeared to express significant power law behavior.

To better understand this outcome, we generated 30 simulated cocktail distributions, based on parameter estimates for the 30 participants and applied the beyond 200 and 3000 and beyond 2.5 SD trimming criteria to the simulated data sets. We then attempted to fit the remaining synthetic observations with the ex-Gaussian, and all 30 data sets were successfully approximated at the *p* ≥ 0.1 level. Similarly, we generated synthetic data sets that entailed compelling power law behavior, with scaling exponents ranging between 2 and 4. We then applied the same exclusive 2.5 SD trimming criteria to the data, and 43% of the distributions were subsequently approximated by the ex-Gaussian using the same *p* ≥ 0.1 criterion described earlier. By contrast, the ex-Gaussian never successfully approximated compelling power law behavior if the 2.5 SD trimming step was omitted.

Clearly the 2.5 SD trimming rule found its utility by facilitating ancillary linear analyses (e.g., Yap et al., 2012). However, the historic rationale for adopting a 2.5 SD rule stems directly from the properties of the tame, symmetric Gaussian distribution function and no contemporary cognitive scientist argues that pronunciation or response time distributions conform to a Gaussian. In any case, the 2.5 SD rule is clearly not appropriate in the context of attempting to distinguish exponential from power law behavior in light of its ability to transform unequivocal power law behavior into apparent exponential behavior.

#### Alternate hazard routines

Response time researchers have repeatedly described typical empirical hazard functions as conforming to one of three categories: They either (1) rise monotonically to an asymptomatic value, (2) rapidly rise to a peak and then decline to an asymptote, or (3) they rapidly rise to a much higher peak and quickly fall off past that point. Taken at face value, the second and third characteristic hazard functions could be consistent with a lognormal or a Wald distribution. Both distributions can produce peaked hazard functions – and the first characteristic hazard function of response time sounds surprisingly consistent with the constant, asymptotic exponential hazard function.

An important caveat must be considered, however. The lion’s share of empirical hazard functions that have appeared in the response time literature used a particular hazard estimation method called the “random smoothing” method, as described by Miller and Singpurwalla (1980). This method uses a smoothing parameter that specifies how many observations to include in each point-by-point hazard estimate. The technique captures hazard function peaks, and works well around the mode of the distribution where observations are plentiful, but it asymptotes to a constant value once the number of remaining observations falls below the value specified by the smoothing parameter.

While the random smoothing method supplies hazard functions that facilitate some types of contrasts, it is challenged in the case of representing decreasing hazard functions. Since the random smoothing method is asymptotically biased to a *constant* hazard function in the context of sparsely populated tails of a distribution, it was not optimal for even-handed comparisons of the hazard function behavior of models that entail exponential or power law tails. This motivated the use of the variable-kernel BIC procedure described earlier in the Method section. The observed shapes of empirical pronunciation time hazard functions varied widely but the kernel-based method revealed their tendency to resemble the ideal cocktail hazard, as peaked and decreasing, an asymptotic pattern that is consistent with power law behavior. Now that the aforementioned statistical issues surrounding distribution fitting, censorship criteria, and hazard estimation are understood, we turn to a stronger test of the generality of the power law hypothesis by applying our suite of statistical analyses to a much larger pronunciation time data set.

## A Test of the Generalizability of the Cocktail Description

The ELP (Balota et al., 2007) maintains a repository of pronunciation times that is available to researchers via the web. A total of 470 participants named two lists of approximately 1500 and 1030 items, respectively, during two experimental sessions. The ELP database includes a vast sample of named items, and a considerable sample of participants, originating from six different universities across the US and Canada. Clearly, it supplies a broad cross-section of word naming performances.

We used these data to determine whether the refined cocktail model could accurately approximate this unusually large sample of participant’s pronunciation time distributions and hazard functions more generally. We were also particularly interested in determining whether the parameters derived for the two different sessions were correlated. The cocktail description is predicated on gaging the dynamics that support cognitive performance and there should be a relationship among the parameters derived from two separate laboratory sessions of a single individual naming different lists of randomly selected words.

### Method

#### Participants and procedure

Four-hundred seventy native English speaking individuals were recruited from a total of six different universities. Each individual received $25 in exchange for their participation. Additional demographic details about the participants are described in Balota et al. (2007).

We applied all the same statistical methods described in the previous experiment to the present data set. We again benchmarked our hazard discrimination routine by replacing, in turn, each of the 470 participant’s empirical pronunciation times with *synthetic* data derived from the best-fit parameters of the cocktail, the ex-Gaussian, and ex-Wald models. Replacing the empirical data with data from known models allowed us to assess the ability of the hazard analysis to discriminate the power law from exponential models.

### Results

#### Distribution fitting outcomes

By the Monte Carlo K–S test standard described above, 420 and 446 of the 470 (89 and 95%) of first- and second-session distributions from the Balota et al. (2007) pronunciation time dataset could be reasonably captured by the cocktail description with *p* ≥ 0.1 – meaning there is at least a 10% chance the empirical distribution *could* have been drawn from the given best-fit cocktail mixture. By contrast, the ex-Gaussian captured 82 and 95 of the 470 (17 and 20%) and the ex-Wald captured 57 and 72 of the 470 (12 and 15%) of the same first- and second-session pronunciation time distributions. The cocktail description captured the vast majority of the individual ELP pronunciation time distributions.

#### Intersession relationships

The estimated parameters for each of the participant’s two sessions were also surprisingly well correlated. The cocktail description successfully approximated both naming sessions for 407 participants. We eliminated one participant’s parameters because a data-gap in his or her distribution’s tail yielded a very large threshold estimate, for a total of 406. Their across-session correlations between lognormal mean and SD (Ω_LN_ and σ) and power law tail-weight parameters (ρ_PL_) were *r*(404) = 0.87, *p* < 0.05, *r*(404) = 0.67, *p* < 0.05, and *r*(404) = 0.64, *p* < 0.05, respectively. The across-session power law scaling exponent (α) correlation was *r*(404) = 0.70, *p* < 0.05. The average session 1 values of the four free refined cocktail parameters were as follows: lognormal mean Ω_LN_, 6.46 (SD = 0.13), lognormal SD, σ, 0.13 (SD = 0.03), scaling exponent, α, 4.67 (SD = 1.08), and proportion power law in the tail, ρ_PL_, 0.42 (SD = 0.13). The average session 2 values of the four free cocktail parameters were: lognormal mean Ω_LN_, 6.45 (SD = 0.14), lognormal SD, σ, 0.13 (SD = 0.03), scaling exponent, α, 4.69 (SD = 1.35), and power law proportion in the tail, ρ_PL_, 0.44 (SD = 0.13).

Overall, the aggregate shape-change between session 1 and 2 corresponded to a tradeoff in which the leading edge, up to and including the mode of the session 2 distributions gained area at the expense of the region just past the session 1 distribution’s mode. A paired sample *t*-test indicated that both the lognormal mean Ω_LN_, and the lognormal SD, σ, were reliably smaller in session 2, *t*(406) = −3.61 *p* < 0.05, *t*(406) = −3.24, *p* < 0.05. The power law proportion in the tail increased reliably, *t*(406) = 3.14, *p* < 0.05, and the scaling exponent, α, did not change reliably across the two sessions. The aggregate shape-change was relatively subtle, and did not identify with particular parameters. It indicated the front end of the session 2 distribution entailed a portion of somewhat faster less variable responses than session 1, but that the slow tails of both distributions were effectively identical. Yap et al. (2012) reported similar outcomes in the context of an ex-Gaussian approximation of the two ELP naming sessions. The robust across-session relationships among the parameters suggest that the refined cocktail model may provide researchers with a useful descriptive tool that assesses individual differences in the dynamic interactions that support performance.

#### Hazard function contrasts

The cocktail hazard captured the majority of the pronunciation time hazard functions for the session 1 data. The cocktail hazard function provided the best description of 329 of the 470 (70%) of the empirical hazard functions. By contrast, the ex-Gaussian and ex-Wald hazard functions best approximated 12 and 18% of the empirical hazard functions, respectively. Thus, 70% of the distributions were classified as consistent with the cocktail description and 30% could be described as consistent with an exponential description. The outcome for the session 2 analysis was similar, 312 of 470 (67%) of the empirical hazard functions were best described by the cocktail description. The ex-Gaussian and ex-Wald distributions best captured 13 and 20% of the empirical hazard functions, respectively.

**Figure 4:**
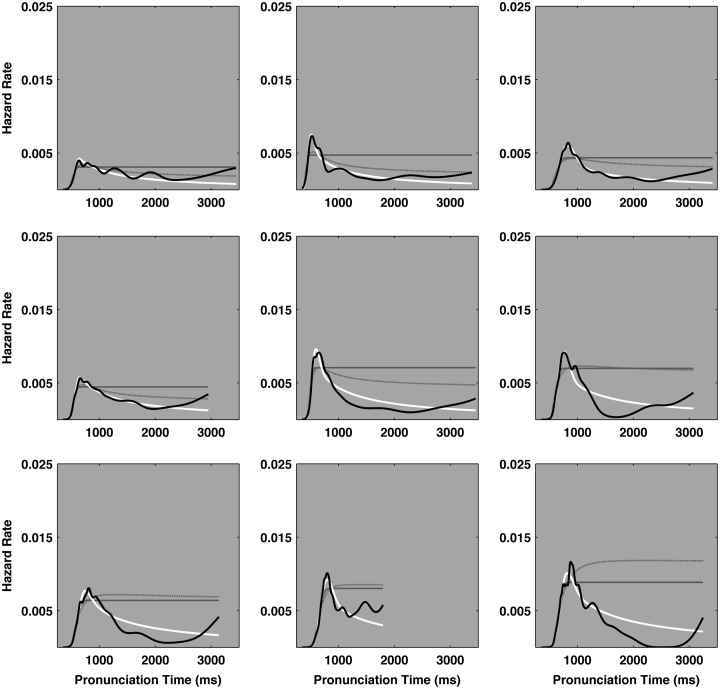
**A depiction of the hazard functions for a random sample of nine participants from the session 1 ELP naming data set**. The *X*-axes in each plot track pronunciation time and the *Y*-axes indicate the hazard rate. The heavy black line represents the empirical hazard functions for each of the nine participants. The ideal cocktail hazard functions are depicted as heavy white lines. The ex-Gaussian and ex-Wald hazard functions are depicted as lighter solid gray and dashed-gray lines, respectively. The case that appears to depict just one solid gray line indicate the ex-Gaussian and ex-Wald hazard functions were effectively identical. The majority of the hazard functions entail a peak and their slow tails more closely match the power law decay indicated in the cocktail description than the exponential decay indicated by either the ex-Gaussian or ex-Wald models. The plots are depicted according to the ascending rank order of the scaling exponent derived from the cocktail fits. That is, the distribution in the top left plot yielded the smallest power law scaling exponent (α = 3.74), the bottom right plot yielded the largest scaling exponent (α = 8.01). α = power law scaling exponent.

**Figure 5:**
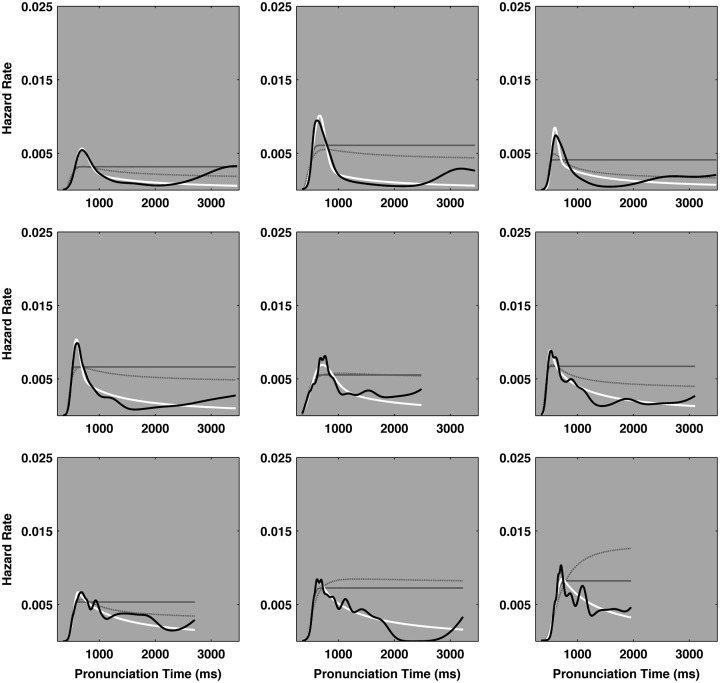
**The hazard functions for a random sample of nine participants from the Session 2 ELP naming data set**. The *X*-axes in each plot track pronunciation time and the *Y*-axes indicate the hazard rate, in events per millisecond. The heavy black line represents the empirical hazard functions for each of the nine participants. The ideal cocktail hazard functions are depicted as heavy white lines. The ex-Gaussian and ex-Wald hazard functions are depicted as a lighter solid gray and dashed-gray lines, respectively. The case that seems to depict one solid line indicates the ex-Gaussian and ex-Wald hazard functions were nearly identical. The majority of the hazard functions entail a peak and their slow tails more closely match the power law decay indicated in the cocktail description than the exponential decay indicated by either the ex-Gaussian or ex-Wald models. The plots are depicted according to the ascending rank order of the scaling exponent derived from the cocktail fits. The distribution in the top left plot yielded the smallest power law scaling exponent (α = 3.04), the bottom right plot yielded the largest scaling exponent (α = 7.33). α = power law scaling exponent.

It is notable that the ex-Wald faired better than the ex-Gaussian in the context of the hazard function analysis. The ex-Wald expresses peaked hazard functions that better resemble empirical pronunciation time hazard functions. However, the ex-Wald faired worse than the ex-Gaussian in the context of the density estimation analysis. Substituting a Wald distribution for the Gaussian worsened the match with empirical densities, but improved subsequent hazard performance. Figures 4 and 5 depict a different random selection of 9 participants from the 470 participants that completed the two sessions.

#### Benchmarking simulations

Hazard analyses of synthetic versions of the distributions revealed nearly identical hit rates and false alarm rates across the two sessions. For the first session, when a cocktail description was inserted as the true model the hit rate was 72% and the exponential false alarm rate was 28% (ex-Gaussian and ex-Wald combined), yielding a *d*′ of 1.17. When the ex-Gaussian was the true model, the exponential hit rate was 95% and the cocktail false alarm rate was 5% yielding a *d*′ of 3.29. When the ex-Wald was the true model the hit and false alarm rates were 97 and 3%, respectively, yielding a *d*′ of 3.76.

Similarly, for the second-session, the synthetic fitting exercises indicated a hit rate of 70% when the cocktail description was the true model, and a 30% an exponential false alarm rate, for a *d*′ of 1.04. When the ex-Gaussian was the true model, the exponential hit rate was 96% and the cocktail false alarm rate was 4% yielding a *d*′ of 3.50. The hit and false alarm rates, and thus *d*′ were identical when the ex-Wald was the true model.

Overall, the BIC hazard routine appeared reasonably capable of discriminating power law behavior from exponential behavior, at least in the context of the Experiment 2 Holden et al. (2009) and the Balota et al. (2007) pronunciation time data sets. The BIC hazard analysis was more likely to misclassify the cocktail description as an exponential than to misclassify an exponential as a cocktail description. As such, the direction of the statistical bias in the routine favored the exponential hypothesis over the power law hypothesis. It is also notable that, given the misclassification bias in favor of the exponential hypothesis, the 70 and 67% cocktail hit-rates for the empirical distributions would be plausible outcomes if all the empirical distributions originated from a mixture of samples from lognormal and power law distributions.

### Discussion

The empirical distribution of an individual participant is in some sense the least-common denominator of a successful description of pronunciation time distributions. These analyses indicate that the refined cocktail model successfully describes the empirical pronunciation time distributions for a large majority of individual participants. While the refined cocktail model is an idealized description of pronunciation time distributions, it successfully captures the descriptive statistics of pronunciation time distributions, their probability density functions, and their hazard functions. It also captures the qualitative differences across individual participants that typically emerge in naming performance. For instance, faster more skilled participant’s distributions are often accommodated by a majority of lognormal samples, less skilled participant’s distributions can be largely approximated by a majority of inverse power law samples, and straightforward mixtures of lognormal and power law accommodate behavioral profiles that span those two extremes.

It is perhaps surprising that the exponential models faired so poorly: Could there have been an error in fitting the distributions? The essential fact is that success for the ex-Gaussian was completely contingent on implementing 2.5 SD truncation operation. For instance, we applied the more exclusive trimming criteria associated with standard ex-Gaussian analyses by deleting observations beyond 200 and 3000 ms, and then deleting responses beyond 2.5 SD from the mean (deleting altogether about 35,000 additional observations). Once applied, the ex-Gaussian then captured 329 (70%) and 349 (74%) of the first- and second-session distributions, 301 and 267% increases, respectively. The ex-Wald yielded more modest improvements.

The hazard plots clarify how the basis of the poor exponential fit does not result exclusively from the observations in the distribution’s tails (e.g., Figures 4 and 5). The exponential models are unable to *simultaneously* capture both the typically high hazard rates around the empirical distribution’s modes and the much lower hazard rates that typically show up in the distribution’s tails. In short, the exponential model’s variability is too homogeneous to accurately depict both aspects of the empirical distributions. Excising the observations in the tails removes the low hazard rates in the tails that are difficult for the exponentials, and results in a higher overall empirical hazard rate. This, in turn, facilitates the exponential model’s ability to approximate the high empirical hazard rates around the empirical distribution’s modes, leading to the appearance of better performance for the exponentials.

Truncation procedures target anomalous data. Here we confront something quite different: The bulk of the participants routinely express extreme observations, lawfully consistent with a power law. Once those observations are systematically excised, only then can the exponential plausibly mimic the empirical patterns. Are some of those observations contaminants, such as voice key failures, lapses in attention, working memory, or even scratching an itch during the experiment? Perhaps, but it is implausible to claim that such a large contingent of the measurements, all systematically located on the distributions’ extrema, are exclusively contaminants that must be ignored in the course of scientific analysis.

In any case, the contaminant hypothesis seeks to displace a theoretically motivated account with a vague place-holder, and must bear the burden of proof: What unsystematic contamination reliably mimics power law behavior so well it is mistaken for it on hundreds of occasions? Offering up a host of unsystematic factors, perhaps unrelated to naming, that align themselves, by coincidence, as a power law would seem to contradict the laws governing randomness – the Central Limit theorem indicates a Gaussian in that case. By contrast, interaction dominant dynamics supply a straightforward explanation of the shape of the empirical distributions.

#### Model selection

The BIC model-contrasting conventions are most useful and appropriate in the context of commensurate modeling frameworks – models that share formative assumptions but that differ in their details. For instance the ex-Gaussian and ex-Wald share the assumption of a characteristic scale and the component dominant dynamics of information accrual. The cocktail description, however is hypothesized to portray interaction dominant dynamics associated with emergence and self-organization.

The cocktail description prevailed in the probability density and hazard analyses, despite the strict BIC penalty imposed in the hazard analysis. Of course, there are any number of ways to evaluate models against each other. A natural concern regarding our BIC contrasts is they might be biased against the ex-Wald, given a fourth parameter was added to the model. However, our density estimation procedures did not penalize parameters, and the ex-Wald, nevertheless, captured only a small minority of the cases.

Most important, the *d*′ analyses on the parameters from the Balota et al. (2007) ELP data indicated that the effect of imposing the BIC penalty actually reduced the likelihood of deciding in favor of the cocktail description, not the ex-Wald or ex-Gaussian. This was the case even when the cocktail was explicitly specified in simulations as the *true* model. So, at least in the context of deciding between the exponential and power law functions presented here, the popular conventional BIC approach to model discrimination has a potential to hobble rather than facilitate accurate model selection (cf., Gilden, 2009). These facts underscore the critical role that a theoretical context can play in the evaluation of statistical outcomes (Van Zandt and Ratcliff, 1995).

**Figure 6:**
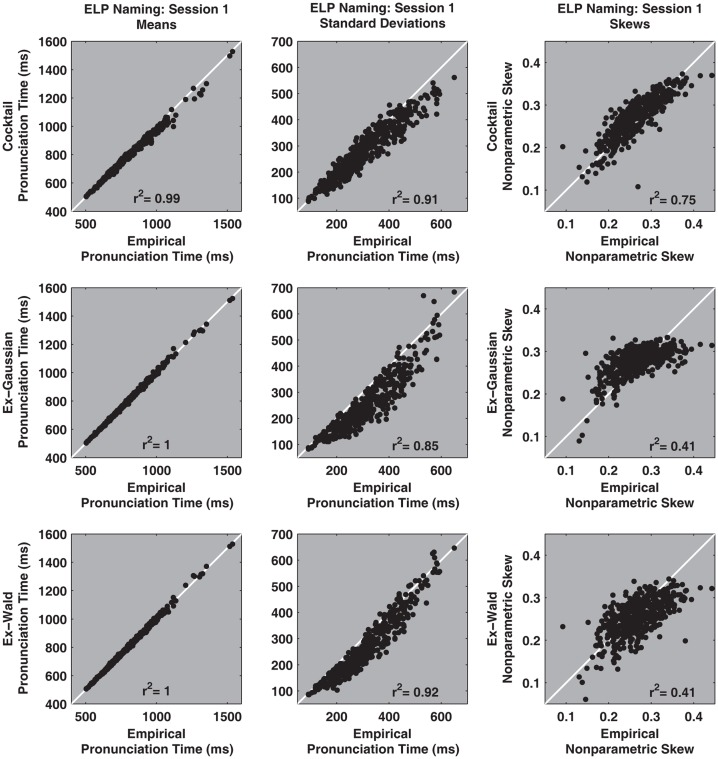
**The *X*-axis of the top three scatter plots depict estimates of the empirical distribution’s mean, standard deviation, and non-parametric skew against same statistics taken from one realization of a synthetic cocktail distribution (*Y*-axis), using the parameters for each of the 470 session 1 participants**. The second and third rows depict the same contrasts for the ex-Gaussian and e-Wald distributions, respectively. Overall, the cocktail description recovers descriptive parameters better than the exponential alternatives. The estimates were computed for all participants, regardless of whether the fits were reliable or not.

**Figure 7:**
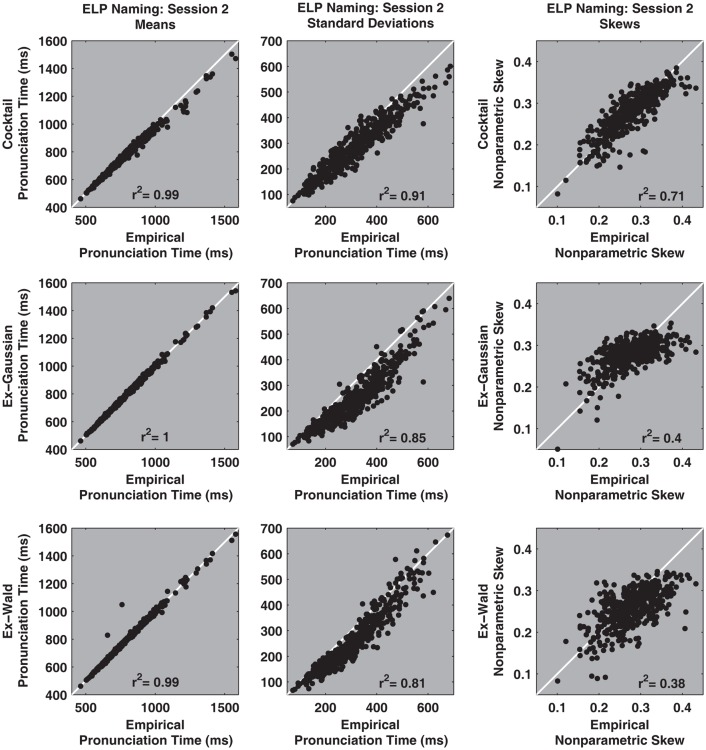
**The *X*-axis of the top three scatter plots depict estimates of the empirical distribution’s mean, standard deviation, and non-parametric skew against same statistics taken from one realization of a synthetic cocktail distribution (*Y*-axis), using the parameters for each of the 470 session 2 participants**. The second and third rows depict the same contrasts for the ex-Gaussian and e-Wald distributions, respectively. Overall, the cocktail description recovers descriptive parameters better than the exponential alternatives. The estimates were computed for all participants, regardless of whether the fits were reliable or not.

The cocktail description characterizes the details of pronunciation times distributions more faithfully than either the ex-Gaussian or ex-Wald alternatives. Figures 6 and 7 illustrate how the cocktail description recovers standard descriptive statistics of individual participant’s pronunciation time distributions better than either the ex-Gaussian or ex-Wald distributions.

All the functions faithfully recover the distribution’s means. However, relative to the cocktail and ex-Wald, the ex-Gaussian’s ability to recover empirical standard deviations is somewhat degraded. The sum of the ex-Gaussian’s mean and exponential rate parameters is equal to the distribution’s average value. This and other built-in constraints may degrade the ex-Gaussian’s ability to characterize descriptive statistics for distributions that are not true ex-Gaussians (Schmiedek et al., 2007; Van Zandt, unpublished manuscript). By contrast, the cocktail distribution’s free parameters are not similarly constrained.

Non-parametric skew indexes the inter-relationships among three common descriptive statistics, the mean, median, and standard deviation. Only the cocktail description is reasonably capable of mirroring the empirical measures of non-parametric skew ([M − Md]/SD). By contrast, both exponential models generally fail to successfully recover this statistic. The scatter plots also demonstrate that the exponential models systematically underestimate the skew of the empirical distributions.

The refined cocktail description successfully accounted for both the 1100-trial pronunciation time distributions described in Experiment 2 of Holden et al. (2009) and the 1500- and 1030-trial pronunciation time distributions described by Balota et al. (2007). In each case the empirical distributions were composed of large numbers of observations. In each case the cocktail function supplied a better approximation of both the participants’ distributions and hazard functions than the ex-Gaussian or ex-Wald alternatives – but specific instantiations of models are highly malleable.

The more important statistical outcome was the general support for the power law hypothesis over the exponential hypothesis. On one hand, this finding buttresses the cocktail description as a plausible and sufficient, but not necessary description of pronunciation time distributions. On the other hand, it renders any model rooted in assumptions consistent with an exponential process as insufficient, and therefore suspect.

The weight of the presented evidence suggests that power law decay is a better description of the slow, heavy tails of pronunciation time distributions than exponential decay. Power laws are symptomatic of scale-free systems. The finding in favor of the power law suggests a perspective on cognitive performance that is not broadly subscribed to in the discipline.

## General Discussion

The cocktail description of pronunciation times was motivated within a framework supplied by complexity science. Studies concerning a wide range of complex physical systems repeatedly revealed generic patterns of interaction among a system’s components that transcend the details of the system components themselves (Nicolis, 1989; Bak, 1996; Jensen, 1998). Complexity scientists uncovered and articulated a characteristic mode of interaction among interdependent system components called *interaction dominant dynamics*. Under specific circumstances, system elements that entail interdependent reciprocal (feedback) couplings are capable of coordinating their behavior in a manner that gives rise to specific emergent patterns (e.g., Yates, 1987; Bak, 1996; Jensen, 1998; Walleczek, 2000; Camazine et al., 2001). Among other things, interaction dominant dynamics are associated with power law event-time distributions and long-term fractal 1/f patterns of temporal correlation; patterns and phenomena that are widely observed in pronunciation time and other response time performances (e.g., Gilden, 2001; Van Orden et al., 2003a, 2005; Kello et al., 2008, 2010; Holden et al., 2009; for an overview, see Turvey and Moreno, 2006).

An inverse power law distribution is symptomatic of coordinative interdependence among the processes that give rise to discrete events. The relationship between any given interval on the *X*- and *Y*-axis of an ideal power law probability density is proportional to the whole probability density – it is scale invariant and self-similar. Thus, no event duration or magnitude is typical. Instead, the size of measured events may span many orders of magnitude. In physical and biological systems the lack of a characteristic scale is symptomatic of interaction dominant dynamics among the processes and constraints that compose complex systems.

As we mentioned in our introduction, speech is known, via *independent evidence*, to be governed by coordinative synergies (Kelso et al., 1984). Coordinative synergies imply the presence of multiplicative and reciprocal dynamics that are expressed as a mixture of lognormal and power law samples. Word naming is a speech act, and the distribution and hazard analyses revealed that pronunciation times do reasonably conform to the predicted mixture. Apparently, we have established a fundamental link between the continuous interaction dominant dynamics of word recognition, speech production, and the characteristic shapes of pronunciation time distributions. This narrative regarding cognitive performance is largely consistent with the basic axioms of connectionist modeling, broadly construed (e.g., Farmer, 1990).

### Lognormal stability versus power law flexibility

Appropriately tuned connectionist systems that embrace or mimic the design principles of proportional amplification and recurrent feedback should express a continuum of distributions that resemble the cocktail description. In the past, however, the connectionist enterprise typically relied on linear statistical methods to test predictions, whereas distributional analyses have revealed non-linear dynamics (Montroll and Shlesinger, 1982; West and Deering, 1995; Bak, 1996).

The lognormal shape represents a special case of interdependent dynamics in which the interdependencies among processes are minimized or absent. Just as the *sum* of many independent random variables yields a Gaussian distribution, the *product* of many independent random variables yields a lognormal distribution (Koch, 1966; Ulrich and Miller, 1993). The generic dynamics of two classes of connectionist models illustrate this point. The dynamics of an exclusively *feed-forward* neural-network are governed by the products of all the component artificial neuron’s states and their respective weight functions (see Eq. 5, Farmer, 1990; Ulrich and Miller, 1993). The only dynamics expressed under these circumstances are time-independent and result from relatively pure multiplicative relations among the connectionist system’s variables. Assuming the network variables entail some random noise and span a sufficient range of values, then the distribution of network activation outputs will approximate a lognormal distribution in the long run.

The key to the emergence of a lognormal is the presence of multiplicative or proportional operators linking a system’s variables (e.g., autocatalytic growth). Thus, lognormal distributions are common in chemical and biological systems. For instance, many viral infection incubation-times approximate a lognormal distribution because symptom onsets are linked to the proportional viral growth-rates expressed in the host (Nishiura, 2007).

Adding feedback connections, or *recurrent* dynamics, to a generic feed-forward connectionist system yields a potential for more complex behavior. For instance, an appropriately tuned recurrent system is capable of maintaining a given state across many successive iterations, but a modeler may also tune it to display oscillatory behavior, or even to change its own dynamics over time, as when Hebbian or other learning rules are introduced (e.g., Hinton and Sejnowski, 1986). Adding dynamic complexity naturally yields more potential behavioral states. The increased complexity of recurrent relations give rise to the potential expression of an inverse power law distribution (Kello et al., 2011). This is why the lognormal and power law distributions are conceived as two idealized poles of a continuum in the cocktail model. At one end is exclusively lognormal behavior, at the other end of the continuum is unadulterated power law behavior.

So-called model “small-world” networks, when configured appropriately, approximate a continuum of output behaviors that range from pure lognormal behavior, lognormal-power law mixtures, and pure power law behavior, the same continuum approximated by the cocktail description. The distribution of a network’s outputs depend on the nature of the links among the network’s nodes. Minimizing feedback and interdependence by minimizing the number and extent of the links across a network’s nodes a yields a lognormal distribution of outputs. Progressively increasing number and extent of the links approximates lognormal distributions with power law tails. Once either or both variables pass a critical boundary point, the output distributions exhibit a phase transition and express clear power law behavior (e.g., see Souma et al., 2001; Garlaschelli and Loffredo, 2004). We speculate that complex self-tuning biological systems, such as human beings, may likewise exhibit a phase transition from the more stable lognormal state to the more flexible power law state, and vice-versa, in accord with available constraints (Van Orden et al., 2003a; Holden et al., 2009, 2011). However, extant word recognition models, connectionist or otherwise, rarely exploit or successfully mimic the multiscale interdependence implied in interaction dominant dynamics.

**Figure 8:**
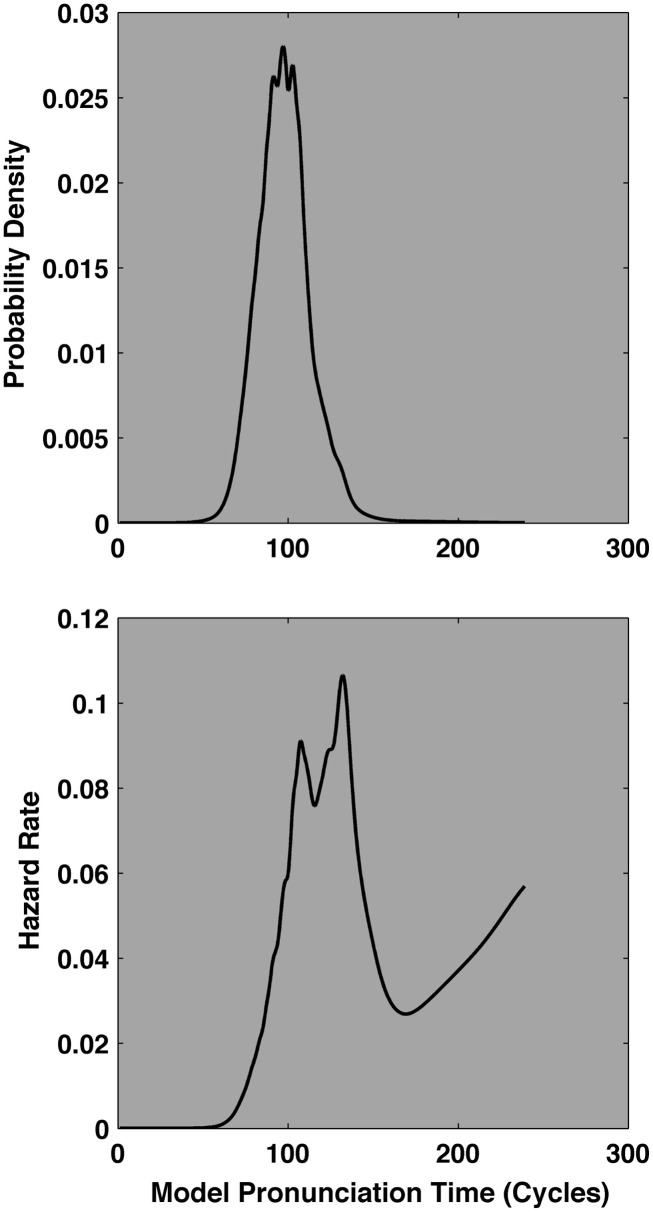
**The top plot depicts the probability density function of the synthetic pronunciation times for the CDP++ model (*N* = 32,263, personal communication, Conrad Perry, April 29, 2012)**. The bottom plot depicts the hazard function for the same data set. The multiple modes in the probability density and the distinct peaks in the hazard function seem to indicate the model expresses distinct processing regimes and very stable dynamics.

Instead, most connectionist word recognition models emphasize additive and/or extremely stable multiplicative dynamics, and they tend to express characteristic time scales of interaction (i.e., they lack power law behavior). For instance, Figure 8 depicts the hazard function for more than 32,000 synthetic pronunciation times derived from the Connectionist-Dual-Process model (CDP++) a successful large-scale model of reading aloud (Perry et al., 2010). In this plot the *X*-axis depicts an analog of pronunciation time, the number cycles the model required to output a pronunciation. The CDP++ probability density appears to have three distinct modes. It is also very nearly symmetrical, with a non-parametric skew of 0.069, less than any of the empirical distributions. Overall, the CDP++ model yields a hazard function consistent with (at least) two distinct regimes: stable, low-variability Gaussian or lognormal that carries with it the bulk of the cognitive effects observed in naming, and a delayed more variable regime that exhibits peaks consistent with (but not exclusive to) a lognormal. The model yielded pronunciations for the bulk of its lexicon (76%) by the first hazard function peak, and it pronounced 98% by the second peak. In fact, for this model, the bulk of the benchmark mean pronunciation time effects such as consistency, frequency, and length, unfold prior to the first hazard peak, in the context of a sharply increasing hazard rate that resembles the increasing hazard function of a Gaussian, and is symptomatic of a characteristic time-scale of interaction; this pattern is largely consistent with component dominant dynamics. However, as we demonstrated, the empirical distributions overwhelmingly indicate the presence of interaction dominant dynamics. Put differently, the model’s behavior reveals the dynamics of relatively independent processes, the empirical data reveals the dynamics of relatively interdependent processes.

As we illustrated, human pronunciation time hazard functions are qualitatively different, their shapes vary widely across a broad continuum, ranging from strongly peaked to barely peaked – a spectrum of qualitative differences consistent with a continuum ranging from lognormal to power law behavior. Nevertheless, despite where an individual fell on this continuum, they had accurately pronounced the bulk of the presented items. Extant word recognition models capture only a slice of this continuum and tend to reify it as the activity of isolable cognitive processes, such as lexical processing (e.g., Rayner and Pollatsek, 1989; Seidenberg and Plaut, 1998; Perry et al., 2010; see also Van Orden et al., 2001). This is because models are designed with an eye toward capturing *mean* effects, and differences in means are interpreted to indicate the distinct time-courses of encapsulated processing – thus theory effectively recapitulates method.

An alternate hypothesis motivated by the presence of lognormal and power law mixture distributions is that word naming dynamics rely on many nested and reciprocally coupled time scales of interaction. For example, eye movement dynamics, such as saccades, unfold on very fast time scales, and are themselves apparently organized in interaction dominant dynamics (Stephen and Mirman, 2010). Dynamics are nested all the way down to the ultimate time scales of neural and axonal firing and the immediate mechanical time scales of articulation (e.g., Bassingthwaighte et al., 1994; Turvey, 2007). Similarly, the presence of 1/f noise (long-range correlation) in trial-series of pronunciation times indicates that faster time-scale dynamics supporting individual pronunciations are themselves nested within slower time-scale processes that also constrain the pronunciation of printed words (e.g., Van Orden et al., 2003a; Holden et al., 2009). Next, we examine some potential implications for interpreting the impact of linguistic and cognitive effects in naming performance, as viewed through the lens of standard linear analyses versus coupled dynamics, nested across multiple time scales.

### Multiple time scales of cognition and action

Conventional component dominant word naming models place perceptual, cognitive, and articulatory factors in sequentially ordered and encapsulated stages, spanning the interval between stimulus and response (e.g., Rayner and Pollatsek, 1989), but this narrative yields apparent paradoxes. For example, Spieler and Balota (1997) used multivariate regression analyses to show that phonemic and articulatory factors capture more than twice as much unique item-level pronunciation time variance than the sum of cognitive predictors such as word frequency, length, and Coltheart’s *N* (e.g., 30 versus 13%, Table 4, p. 414). Similarly, Balota et al. (2004) attribute 39% of younger participant’s item-level naming variance to onset phonemes but only about 10% to lexical characteristics (Table 6, p. 299). Why do cognitive factors capture so little variance?

At first glance, a pronunciation time appears to be sampled at the transition point between the stabilization of cognitive dynamics and the onset of articulatory dynamics. However, as our introduction explains, a complex coordinative articulatory dynamic must be largely in place before a participant can even utter a word’s onset. This requires, as well, that cognitive and perceptual constraints are largely in place to support more slowly unfolding articulatory constraints (and consequent acoustics).

Yet a regression analysis assumes all factors entail an equivalent potential to impact variability. However, consider a regression analysis conducted on the face value sum-totals of three coins, repeatedly and randomly sampled from the set: penny, nickel, dime, quarter, and half-dollar. If positive and negative face values are equiprobable, then a predictor that perfectly tracks the inclusion of the penny in the sums would nevertheless correlate poorly with the sum-totals. Since the penny’s contribution to the total is tiny, the predictor appears to perform poorly. When time is the dependent measure, predictors tied to faster process behave like the penny and necessarily capture less variability.

The perceptual and cognitive dynamics of word recognition tend to unfold on very fast time scales (e.g., <200 ms, Pulvermüller et al., 1999; Strijkers and Costa, 2011; Strijkers et al., 2011; Experiment 2, Van Orden et al., 1999), faster than corresponding articulatory dynamics. As such, they typically affect smaller amplitude changes in variability. Since time is the dependent measure in speeded naming, slower time-scale dynamics necessarily have a larger sway in affecting variability. By definition, they generate larger amplitude changes in variability. Thus, theoretical debates framed in terms of the percent of variance captured are potentially misleading.

Only occasionally are faster time-scale dynamics able to reach out and “touch” more slowly unfolding dynamics. That is, when faster time-scale dynamics fail to stabilize and enfold with slower time-scale dynamics, they may perturb the trajectory at the slower time-scale. As when a participant has difficulty resolving the pronunciation of items with ambiguous vowel pronunciations relative to items with invariant vowel pronunciations (e.g., feed-forward and feedback consistency: Stone et al., 1997; Gottlob et al., 1999; Holden, 2002; Balota et al., 2004; Van Orden and Kloos, 2005), or items with less constrained, more ambiguous onsets versus those containing more constrained onsets (e.g., *sin* versus *spin*, Kawamoto and Kello, 1999).

In samples of printed text, letter-phoneme relations recur more often than spelling-body pronunciation-rime relations, that themselves recur more frequently than specific whole-words. This rank order approximates the relative time scales of the perceptual and cognitive dynamic coherence for these respectively nested sets of relations, from faster to slower (Van Orden and Goldinger, 1994; Holden, 2002). The faster the dynamics stabilize, the less variability they will contribute to the pronunciation time. Thus, while consistency effects in the relations between spelling patterns and pronunciations are nearly always present in large-scale multiple regression analyses of pronunciation times, they tend to account for relatively little variance, compared to word frequency, for instance.

Furthermore, a coherent dynamic at the scale of whole-words may simultaneously amplify the stability of the sub-word relations and facilitate the coherence of constraints for articulatory dynamics. For example, frequent words are associated with shorter onset durations (Kawamoto et al., 1999). More generally, speeded word naming emphasizes strongly constrained, largely unique relationships between whole-word patterns of spelling and whole-word pronunciations. These relations are so engrained in an individual’s linguistic experience that the dynamic supporting the pronunciation of both frequent and less frequently encountered items is relatively stable, and yields only a modest frequency “effect,” relative to lexical decision, for instance (Van Orden and Goldinger, 1994). Perceptual and cognitive dynamics unfold on the relatively faster time scales of the nervous system. By contrast, articulatory dynamics unfold on the slightly slower time scales of physiology; they require appropriate changes in the physical configuration of muscles and vocal tracts and take more time to cohere, hence their advantage in regression analyses.

## Conclusion

Tasks, manipulations, and linguistic environments tend to emphasize different aspects of a participant’s dexterity – the fluency of their perceptual, cognitive, and behavioral skills. Cooperative participants can be relied on to opportunistically optimize their performance to best serve the experimenter assigned goals (Van Orden and Holden, 2002; Van Orden et al., 2003a). The variables that crucially support successful performance in a given context will tend to dominate that performance, and give rise to “effects” that are conditional on, or interact with the particulars of the task at hand. As such, small changes in instructions, the laboratory context, or other relevant circumstances may yield dramatic changes in the measured behavior (Van Orden et al., 1999; Kloos and Van Orden, 2005; Castillo et al., 2011; Holden et al., 2011).

Dynamics apparently ancillary to cognition, such as response velocity, are nevertheless measurably influenced, via interaction dominant dynamics, by more stable versus less stable faster time-scale dynamics. For example Abrams and Balota (1991) reported increased response velocity was associated with high frequency items in lexical decision. Even limb and postural trajectories are measurably, but subtly influenced by cognitive manipulations (Moreno et al., 2011). Similarly, frequency manipulations impact word naming performance even after lengthy response delays are imposed (Goldinger et al., 1997). These findings supply a different kind of corroboration of interaction dominant dynamics, they highlight unanticipated dynamic couplings across the spectrum of heterogeneous processes of body and mind that support cognitive performance.

Apparently, speeded word naming performance relies on interdependent, and malleable coordinative dynamics. The parameters of the cocktail description offer an abstract and indirect assessment of those dynamic states. Rather than referring to specific cognitive operations, stages, or processes, they instead estimate the aggregate stability of the emergent coordinative activity that self-organizes to support speaking words aloud.

This portrait specifies the challenge in skilled speeded naming performance as achieving a functional organization that approximates a characteristic scale. This is accomplished by dynamically enfolding environmental, perceptual, cognitive, and neuromuscular constraints that, themselves, span a range of time scales. The transition from unskilled to skilled naming performance entails a qualitative compression of degrees of freedom that reflects a bifurcation or phase transition from power law behavior to lognormal behavior. Likewise, laboratory manipulations that increase functional ambiguity will tend to destabilize performance and, past a critical point, invite the expression of power law behavior (Van Orden et al., 1999; Holden, 2002; Holden et al., 2009).

As we mentioned, coordinative synergies support articulation by striking reciprocal and adaptive coupling among processes spanning a range of time scales. In many natural systems this form of interdependence amounts to a recipe for self-organization. Thus, it is plausible to propose that pronunciation time measurements reflect the same kinds of dynamics that support self-organization in other natural systems.

## Conflict of Interest Statement

The authors declare that the research was conducted in the absence of any commercial or financial relationships that could be construed as a potential conflict of interest.

## Appendix

### Mathematical formulation of the lognormal-power law mixture distribution derived by Srinivasan Rajaraman

In this Appendix, the probability distribution function (PDF) of the Holden et al. (2009) cocktail model description is derived. The PDF of the cocktail model is conceived as a mixture of a lognormal and a power law distribution. As such, the cocktail model, *f*(*t*), can be mathematically expressed as follows:

(A1) $$f\left(t\right)=\omega *\text{pdf}\text{\_}\text{LN}\left(t\right)+\left(1-\omega \right)*\text{pdf}\text{\_}\text{PL}\left(t\right),$$

where pdf_LN(*t*) = (1/(*t*$\left(1/\left(t*\sigma *\sqrt{2\pi }\right)\right)*$ exp(− (ln(t) − Ω_LN_)^2^/2σ^2^) and $\text{pdf}\text{\_}\text{PL}\left(t\right)=\left(\alpha -1\right)*{\text{Ω}}_{\text{PL}}^{\alpha -1}*t^{-\alpha },$ *t* ≥ Ω_PL_, and = 0, *t* < Ω_PL_, represent, respectively, a lognormal (Casella and Berger, 2002) and an inverse power law PDF (Clauset et al., 2009). Here *t*, a random variable, denotes the response latency, Ω_LN_ and σ represent, respectively, the mean and the standard deviation of the distribution of the natural logarithm of the variable *t*, whereas α and Ω_PL_ represent, respectively, the scaling exponent and the threshold parameter of the inverse power law distribution, and ω is a parameter, with ω ∈ [0, 1], that denotes the relative proportion of the lognormal distribution in the cocktail model. The aforementioned formulation of the inverse power law PDF is valid only for α > 1.

In this formulation of *f* (*t*), as ω tends to 0, *f* (*t*) tends to a pure inverse power law distribution, whereas as ω tends to 1, *f* (*t*) tends to a pure lognormal distribution. For intermediate values of ω and for any set of feasible values of the parameters Ω_LN_, σ, α, and Ω_PL_, *f* (*t*) discontinuously varies across *t* = Ω_PL_, i.e., the left-hand limit (LHL) and the right-hand limit (RHL) of *f* (*t*) at *t* = Ω_PL_ are always unequal. Therefore, we seek an alternative formulation of the cocktail model. Accordingly, we propose a different mathematical form of the cocktail model as follows

(A2) $$f\left(t\right)=\left\{\begin{array}{cc} \frac{{\text{ρ}}_{\text{FLN}}}{C\left({\text{Ω}}_{\text{PL}}\right)}\text{pdf}\text{\_}\text{LN}\left(t\right),\; & t<{\text{Ω}}_{PL}\\ \frac{{\text{ρ}}_{\text{BLN}}}{1-C\left({\text{Ω}}_{PL}\right)}\text{pdf}\text{\_}\text{LN}\left(t\right)+{\text{ρ}}_{\text{PL}}*\text{pdf}\text{\_}\text{PL}\left(t\right),\; & t\geq {\text{Ω}}_{\text{PL}}, \end{array}\right.$$

where ρ_FLN_, ρ_BLN_, and ρ_PL_ are positive real numbers, with ρ_FLN_ + ρ_BLN_ + ρ_PL_ = 1. Therefore, each ρ_FLN_, ρ_BLN_, and ρ_PL_ is less than or equal to 1. Qualitatively speaking, the PDF of the cocktail model described in Eq. A2 is a pure lognormal PDF ∀*t* ∈ (0, Ω_PL_) and a mixture of lognormal and inverse power law PDFs ∀*t* ∈ (Ω_PL_, ∞). Here, ρ_FLN_ denotes the proportion of the cocktail model PDF within the interval *t* ∈ (0, Ω_PL_), referred to as the front end of the cocktail PDF, and 1 − ρ_FLN_ = (ρ_BLN_ + ρ_PL_) denotes the proportion of the cocktail model PDF within the interval *t* ∈ (Ω_PL_, ∞), referred to as the back end of the PDF. Further, the back end of the cocktail PDF is a mixture of lognormal and power law distributions with ρ_BLN_ and ρ_PL_, respectively, representing their mixture proportions. Consequently, both ρ_BLN_ and ρ_PL_ are always ≤1 − ρ_FLN_. That is, when ρ_BLN_ = 1 − ρ_FLN_, ρ_PL_ = 0, and vice-versa. Here, $C\left({\text{Ω}}_{\text{PL}}\right)=\int_{t=0}^{{\text{Ω}}_{\text{PL}}}\text{pdf}\text{\_}\text{LN}\left(t\right)\;dt$ denotes the cumulative density of the lognormal distribution at *t* = Ω_PL_.

The advantage of the reformulated cocktail model PDF, given in Eq. A2, is that we can achieve the continuity of both *f* (*t*) and its first derivative ∀*t* ∈ (0, ∞). Hereafter, smoothness of *f* (*t*) implies the continuity of the first derivative of *f* (*t*). In particular, we need to achieve the smoothness of *f* (*t*) only at *t* = Ω_PL_, as *f* (*t*) is both continuous and smooth otherwise. In the following section, we show what constraints are necessary to ensure the continuity and smoothness of the proposed cocktail model PDF at *t* = Ω_PL_.

#### Establishing continuity and smoothness of the cocktail model PDF

To achieve the continuity of the cocktail model PDF *f* (*t*) at *t* = Ω_PL_, we require that LHL and RHL of *f* (*t*) at *t* = Ω_PL_ be equal. For simplicity, let *A*(*t*) denote pdf_LN(*t*), then the equality between the LHL and RHL of *f* (*t*) at *t* = Ω_PL_ can be expressed as follows

(A3) $$\begin{array}{c} \frac{{\text{ρ}}_{\text{FLN}}}{C\left({\text{Ω}}_{\text{PL}}\right)}A\left({\text{Ω}}_{\text{PL}}\right)=\frac{{\text{ρ}}_{\text{BLN}}}{1-C\left({\text{Ω}}_{\text{PL}}\right)}A\left({\text{Ω}}_{\text{PL}}\right)+{\text{ρ}}_{\text{PL}}\frac{\left(\alpha -1\right)}{{\text{Ω}}_{PL}},\;\\ \left[\frac{{\text{ρ}}_{\text{FLN}}}{C\left({\text{Ω}}_{\text{PL}}\right)}-\frac{{\text{ρ}}_{\text{BLN}}}{1-C\left({\text{Ω}}_{\text{PL}}\right)}\right]A\left({\text{Ω}}_{\text{PL}}\right)={\text{ρ}}_{\text{PL}}\frac{\left(\alpha -1\right)}{{\text{Ω}}_{PL}}. \end{array}$$

Further, if we seek smoothness of *f* (*t*) at *t* = Ω_PL_, then we require that the left-hand derivative and the right-hand derivative of *f* (*t*) at *t* = Ω_PL_ be equal, which can be mathematically expressed as follows

(A4) $$\begin{array}{c} \frac{{\text{ρ}}_{\text{FLN}}}{C\left({\text{Ω}}_{\text{PL}}\right)}B\left({\text{Ω}}_{\text{PL}}\right)=\frac{{\text{ρ}}_{\text{BLN}}}{1-C\left({\text{Ω}}_{\text{PL}}\right)}B\left({\text{Ω}}_{\text{PL}}\right)-{\text{ρ}}_{\text{PL}}\frac{\alpha \left(\alpha -1\right)}{{\text{Ω}}_{\text{PL}}^{2}},\\ \left[\frac{{\text{ρ}}_{\text{FLN}}}{C\left({\text{Ω}}_{\text{PL}}\right)}-\frac{{\text{ρ}}_{\text{BLN}}}{1-C\left({\text{Ω}}_{\text{PL}}\right)}\right]B\left({\text{Ω}}_{\text{PL}}\right)=-{\text{ρ}}_{\text{PL}}\frac{\alpha \left(\alpha -1\right)}{{\text{Ω}}_{\text{PL}}^{2}}, \end{array}$$

where, *B*(*t*) represents *dA*(*t*)/*dt*. Dividing both sides of Eq. A4 by the corresponding sides of Eq. A3, and assuming ρ_PL_ ≠ 0, and *C*(Ω_PL_) < 1, we obtain

(A5) $$\frac{B\left({\text{Ω}}_{\text{PL}}\right)}{A\left({\text{Ω}}_{\text{PL}}\right)}=-\frac{\alpha }{{\text{Ω}}_{\text{PL}}}.$$

Note that $B\left(t\right)=-\left[\frac{A\left(t\right)}{t}\right]\left[\frac{\left(\log\left(t\right)-{\text{Ω}}_{LN}\right)}{\sigma^{2}}+1\right]\;,$ therefore we can simplify Eq. A5 and obtain the following equation

(A6) $${\text{Ω}}_{\text{PL}}=\exp\left[{\text{Ω}}_{\text{LN}}+\sigma^{2}\left(\alpha -1\right)\right]\;.$$

Equation A6 represents an equality constraint among the parameters Ω_PL_, Ω_LN_, σ, and α that guarantees continuity as well as smoothness of *f* (*t*) in Eq. A2 ∀*t* ∈ (0,∞). Moreover, this equation conveys that for fixed values of Ω_LN_ and σ, Ω_PL_ grows exponentially as a function of α and because, by definition, α > 1, we require Ω_PL_ > exp(Ω_LN_), which is the median of the lognormal distribution. Using Eq. A3 and the equation ρ_FLN_ + ρ_BLN_ + ρ_PL_ = 1 and assuming *C*(Ω_PL_) < 1, we get the following expressions for ρ_FLN_ and ρ_BLN_

(A7) $${\text{ρ}}_{\text{FLN}}=C\left({\text{Ω}}_{\text{PL}}\right)*\left[1-{\text{ρ}}_{\text{PL}}\right]+\frac{C\left({\text{Ω}}_{\text{PL}}\right)*\left[1-C\left({\text{Ω}}_{\text{PL}}\right)\right]*{\text{ρ}}_{\text{PL}}\left(\alpha -1\right)}{{\text{Ω}}_{\text{PL}}*A\left({\text{Ω}}_{\text{PL}}\right)}$$

and

(A8) $${\text{ρ}}_{\text{BLN}}=\left[1-C\left({\text{Ω}}_{\text{PL}}\right)\right]*\left(1-{\text{ρ}}_{\text{PL}}-\frac{C\left({\text{Ω}}_{\text{PL}}\right)*{\text{ρ}}_{\text{PL}}*\left(\alpha -1\right)}{{\text{Ω}}_{\text{PL}}*A\left({\text{Ω}}_{\text{PL}}\right)}\right).$$

Equation A8 implies that, for fixed values of the parameters Ω_PL_ and α, ρ_BLN_ is a decreasing function of ρ_PL_. This is evident from the fact that the back end of the cocktail PDF is shared between the lognormal and the power law distributions; therefore, the proportion of each of the two distributions increases as the proportion of the other decreases. Hence, to ensure that ρ_BLN_ ≥ 0, we require that

(A9) $$0\leq {\text{ρ}}_{\text{PL}}\leq {\left[1+\frac{C\left({\text{Ω}}_{\text{PL}}\right)*\left(\alpha -1\right)}{{\text{Ω}}_{\text{PL}}*A\left({\text{Ω}}_{\text{PL}}\right)}\right]}^{-1}.$$

Substituting the expressions for ρ_FLN_ and ρ_BLN_, respectively, from Eqs. A7 and A8 and for Ω_PL_ from Eq. A6 into Eq. A2, we obtain *f* (*t*) whose values are uniquely determined by the parameters ρ_PL_, α, Ω_LN_, and σ.

### Summary

We summarize the description of the cocktail model PDF, which is defined in the interval *t* ∈ (0, ∞), as the mixture sum of the following three PDFs:

1) $\frac{1}{C\left({\text{Ω}}_{\text{PL}}\right)}\text{pdf\_LN}\left(t\right)\;\forall t\in \left(\text{0,}\;{\text{Ω}}_{\text{PL}}\right)\text{ and 0 }\forall t\in \left[\;{\text{Ω}}_{\text{PL}},\infty \right)$
2) $0\forall t\in \left(0,{\text{Ω}}_{\text{PL}}\right)\text{ and }\frac{1}{1-C\left({\text{Ω}}_{\text{PL}}\right)}\text{pdf\_LN}\left(t\right)\;\forall t\in \left[{\text{Ω}}_{\text{PL}},\infty \right)$
3) $0\forall t\in \left(0,{\text{Ω}}_{\text{PL}}\right)\text{ and pdf\_PL}\left(t\right)\;\forall t\in \left[{\text{Ω}}_{\text{PL}},\infty \right)$,

with ρ_FLN_, ρ_BLN_, ρ_PL_, respectively, their mixture proportions. Hence, *f* (*t*) in Eq. A2 is a legitimate PDF, in that *f* (*t*) ≥ 0 and $\int_{t=0}^{\infty }f\left(t\right)\;dt=1.$

#### Corollaries

1) When ρ_PL_ = 0, ρ_FLN_, and ρ_BLN_ are equal to *C*(Ω_PL_) and 1−*C*(Ω_PL_), respectively.
2) When ${\text{ρ}}_{\text{PL}}={\left[1+\frac{C\left({\text{Ω}}_{\text{PL}}\right)*\left(\alpha -1\right)}{{\text{Ω}}_{\text{PL}}*A\left({\text{Ω}}_{\text{PL}}\right)}\right]}^{-1},$ ρ_FLN_, and ρ_BLN_ are equal to 1 − ρ_PL_ and 0, respectively.

Matlab^®^ code that implements the cocktail model available for download at http://homepages.uc.edu/~holdenjn/.
